# Analysis of Population Structure and Selective Signatures for Milk Production Traits in Xinjiang Brown Cattle and Chinese Simmental Cattle

**DOI:** 10.3390/ijms26052003

**Published:** 2025-02-25

**Authors:** Kailun Ma, Xue Li, Shengchao Ma, Menghua Zhang, Dan Wang, Lei Xu, Hong Chen, Xuguang Wang, Aladaer Qi, Yifan Ren, Xixia Huang, Qiuming Chen

**Affiliations:** College of Animal Science, Xinjiang Agricultural University, Urumqi 830052, China; makailun0829@163.com (K.M.); lixueli1126@163.com (X.L.); shengchaomasicau@163.com (S.M.); zhangmenghua810@126.com (M.Z.); wangdan01100330@163.com (D.W.); q609468041@sina.com (L.X.); chenhong1212@263.net (H.C.); wangxuguang@xjau.edu.cn (X.W.); aqi@xjau.edu.cn (A.Q.); 15136225372@163.com (Y.R.)

**Keywords:** whole-genome resequencing, Xinjiang brown cattle, Chinese Simmental cattle, genetic diversity, population structure, selective signature

## Abstract

This study aims to elucidate the population structure and genetic diversity of Xinjiang brown cattle (XJBC) and Chinese Simmental cattle (CSC) while conducting genome-wide selective signatures analyses to identify selected genes associated with milk production traits in both breeds. Based on whole-genome resequencing technology, whole-genome single nucleotide polymorphisms (SNPs) of 83 Xinjiang brown cattle and 80 Chinese Simmental cattle were detected to resolve the genetic diversity and genetic structure of the two populations, whole-genome selective elimination analysis was performed for the two breeds of cattle using the fixation index (*F*_st_) and nucleotide diversity (θπ ratio), and enrichment analysis was performed to explore their biological functions further. Both breeds exhibited relatively rich genetic diversity, with the Chinese Simmental cattle demonstrating higher genetic diversity than Xinjiang brown cattle. The IBS and G matrix results indicated that most individuals in the two populations were farther apart from each other. The PCA and neighbor-joining tree revealed no hybridization between the two breeds, but there was a certain degree of genetic differences among the individuals in the two breeds. Population structure analysis revealed that the optimal number of ancestors was three when K = 3. This resulted in clear genetic differentiation between the two populations, with only a few individuals having one ancestor and the majority having two or three common ancestors. A combined analysis of *F*_st_ and θπ was used to screen 112 candidate genes related to milk production traits in Xinjiang brown cattle and Chinese Simmental cattle. This study used genome-wide SNP markers to reveal the genetic diversity, population structure, and selection characteristics of two breeds. This study also screened candidate genes related to milk production traits, providing a theoretical basis for conserving genetic resources and improving genetic selection for milk production traits in Xinjiang brown cattle and Chinese Simmental cattle.

## 1. Introduction

Xinjiang brown cattle and Chinese Simmental cattle are excellent dual-purpose breeds independently bred in China. Xinjiang brown cattle are a crossbreed of Xinjiang local Kazakh cattle as the female parent and imported Alatau cattle, a small number of Costello cattle, and Brown Swiss cattle [1]. This breed is known for its tolerance to roughage, resistance to cold weather and adversity, high-quality milk, delicious meat, and ability to thrive on mountain grasslands. It is considered one of the main cattle breeds in Xinjiang for breeding purposes [2]. Since the introduction of Simmental cattle in China at the beginning of the 20th century, it has been crossed with local cattle for many years. Excellent individuals with high-generation improvement have been selected and bred for strong adaptability, rapid growth, good meat quality, high milk production, high milk fat rates, and other exceptional qualities [3,4].

The Yili region features excellent environmental and resource conditions, making it a key breeding ground for high-quality livestock and poultry in Xinjiang and throughout China. With a significant cattle population and strong milk production, the region has developed several local dairy brands, fostering the progress of high-quality development in its dairy industry. The milk production trait is a crucial aspect of livestock economic traits, as the performance and quality of milk directly affect efficiency and costs, ultimately impacting the economic development of the livestock industry [5]. Milk production traits are quantitative traits, including milk yield, milk fat content, milk protein content, milk fat rate, and milk protein rate, which are regulated by major genes and a variety of minor genes, as well as factors such as disease prevention, feed quality, feeding practices, and various environmental factors [6]. Among the selection targets, milk production is the primary focus of cattle production. Identifying functional gene loci and key genetic variations related to milk production traits is crucial for the molecular breeding of cattle in China. With the development of molecular quantitative genetics, advancements in molecular genetic markers and sequencing technology, and the application of molecular markers and marker-assisted selection (MAS), there has been a significant impact on cattle breeding in relation to milk yield traits. To improve the genomic assessment of milk production traits and understand their molecular mechanisms, the identification of relevant genomic regions and candidate genes is essential [7].

Traditional genetic structure analysis of populations by pedigrees may be affected by missing records, making it difficult to draw accurate conclusions [8]. The advancements in molecular genetics, high-throughput sequencing technology, and bioinformatics in recent years have made 2nd-generation molecular marker technology a crucial tool for enhancing livestock and poultry genetics. This technology has facilitated the study of genetic diversity through single nucleotide polymorphisms (SNPs), which have the characteristics of wide distribution, high polymorphism information, easy detectability, and strong genetic stability [9]. Currently, SNP sequencing technology has been utilized in population genetic studies of various cattle breeds, including Jiaxian red cattle [10], Qinchuan cattle [11], Yunling cattle [12,13], and Dabie Mountain cattle [14], among others, which provide valuable insights for analyzing the structure and genetic diversity of cattle populations. Genome-wide selection signal analysis of populations can be used to detect genes associated with milk production traits effectively. Wang et al. [15] identified candidate genes related to disease resistance (*CDH4*, *SIRPB1*, and *SIRPα*) and the endocrine system (*ADCY5*, *ABCC8*, *KCNJ11*, and *KCNMA1*) in Xinjiang brown cattle by selection signature analysis using *F*_st_ and θπ. Ma et al. [16] selected Xinjiang brown cattle and Chinese Simmental cattle with high or low milk somatic cell count to screen differentially expressed genes and differentially expressed metabolites related to milk production performance by RNA-Seq and LC-MS/MS, revealing key candidate genes, iconic metabolites, and molecular signaling pathways for milk production performance of dual-purpose cattle. In the study conducted by Zhang et al. [17], the *F*_st_ method was used to detect structural variation differences between Xinjiang brown cattle, Kazakh cattle, and Brown Swiss cattle populations. Candidate genes related to milk production traits (*PI4K2A*, *ELOVL3*, *ECHS1*, *SCD*, *TCF7L2*, *PNLIPRP2*, *BTRC*, *PLCE1*) were also identified. Using different selection signal methods can not only verify the reliability of each method but also improve the accuracy of gene localization.

Although some milk-producing genes of dairy cattle have been identified through GWAS and other methods, genome-wide selection signal analysis for Xinjiang brown cattle and Chinese Simmental cattle is still lacking. This study utilized whole-genome resequencing technology to analyze SNP data from Xinjiang brown cattle and Chinese Simmental cattle to assess their genomic genetic diversity, genetic relationship matrix, and individual genetic distance. The aim was to gain insight into population structure and genetic relationships. This study analyzed the fixation index (*F*_st_) and nucleotide diversity (θπ ratio) to identify selection signals in two breeds of cattle and identified candidate genes associated with milk production traits. The genetic background and breeding process of Xinjiang brown cattle and Chinese Simmental cattle were further elucidated at the molecular level to provide a theoretical foundation for the conservation of genetic resources and subsequent precision breeding of these two populations.

## 2. Results

### 2.1. Genomic Data Description of Two Breeds

Xinjiang brown cattle resequencing generated an average of 560 million reads with a mean alignment rate of 99.86% and an average sequencing depth of 30.22×. Chinese Simmental cattle resequencing produced an average of 760 million reads, with an average alignment rate of 99.75% and an average sequencing depth of 40.49×.

### 2.2. Detection and Annotation of SNPs in Cattle Genomes of Two Breeds

**Figure 1:**
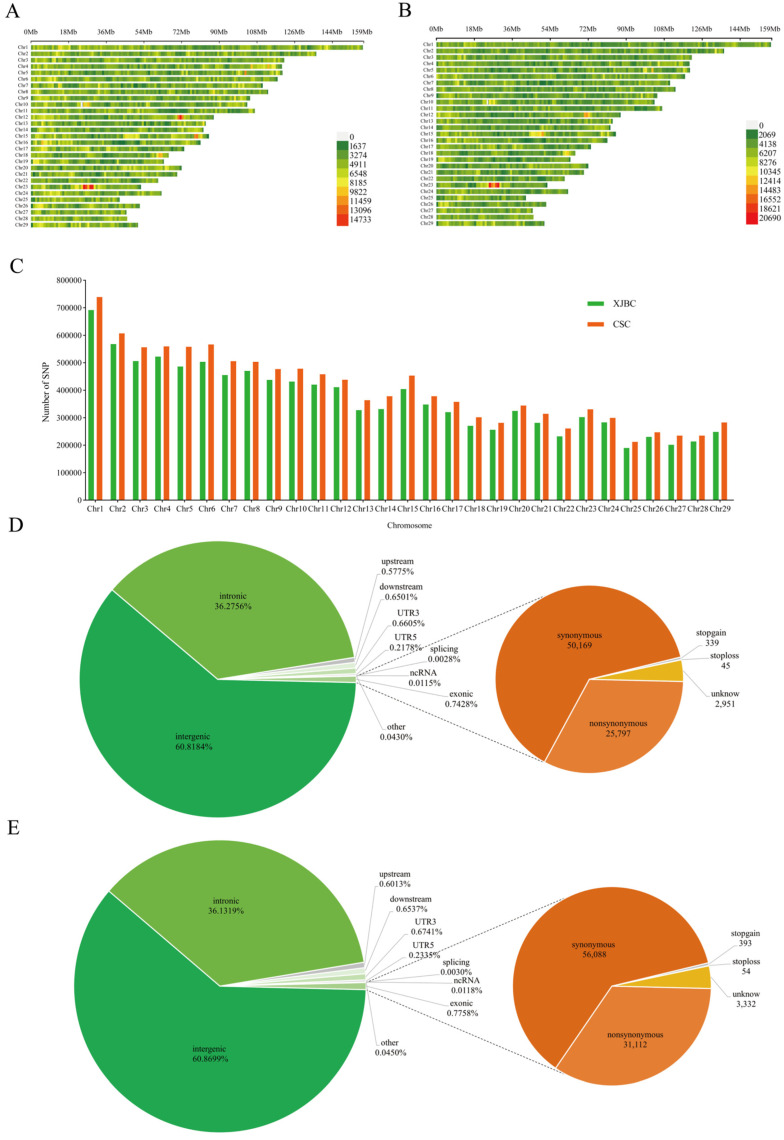
Distribution and functional annotation of SNPs in Xinjiang brown cattle and Chinese Simmental cattle. (**A**) Distribution of SNPs on autosomes in Xinjiang brown cattle. The horizontal coordinates indicate the density or number of SNPs, whereas the vertical coordinates indicate the positions or positional intervals of the 29 chromosomes. Different colors in the figure represent the number of SNPs per 1 Mb range: a color closer to red indicates a higher density of SNPs, a color closer to green indicates a lower density of SNPs, and gray indicates no SNP distribution at that position. (**B**) Distribution of SNPs on autosomes in Chinese Simmental cattle. (**C**) Frequency distribution of SNPs on 29 chromosomes of Xinjiang brown cattle and Chinese Simmental cattle. (**D**) Functional distribution of SNPs in Xinjiang brown cattle. The pie chart to the left displays the distribution of SNPs, including intergenic, intronic, upstream, downstream, UTR3, UTR5, splicing, ncRNA, and exonic regions, based on their location. The pie chart to the right displays the breakdown of exons, including nonsynonymous, synonymous, stop-gain, and stop-loss exons. (**E**) Functional distribution of SNPs in Chinese Simmental cattle.

After GATK variant detection, a total of 10,669,376 high-quality SNPs were retained for analysis in Xinjiang brown cattle, whereas 11,722,337 high-quality SNPs were retained for Chinese Simmental cattle. The density distribution of high-quality SNPs on each chromosome is shown in Figure 1A,B. Figure 1C illustrates the distribution of SNPs on chromosomes in the two populations. Among the autosomes, Chr 1 was the longest (Xinjiang brown cattle: 158.39 Mb; Chinese Simmental cattle: 158.50 Mb) and contained the highest number of SNP loci (Xinjiang brown cattle: 691,843; Chinese Simmental cattle: 738,798); Chr 25 was the shortest (Xinjiang brown cattle: 42.33 Mb; Chinese Simmental cattle: 42.34 Mb) and contained the lowest number of SNP loci (Xinjiang brown cattle: 190,006; Chinese Simmental cattle: 212,032). The two breeds were functionally annotated using ANNOVAR software based on the filtered SNPs (Figure 1D,E). There were 6,488,949 SNPs located in the intergenic region of Xinjiang brown cattle, accounting for 60.8% of the total number of SNPs. There were 3,870,384 and 79,249 SNPs located in the intronic and exonic regions, accounting for 36.3% and 0.7% of the total number of SNPs, respectively. Within the exon region, there were 25,797 nonsynonymous mutations and 50,169 synonymous mutations. In Chinese Simmental cattle, 7,135,370 SNPs were located in the intergenic region, accounting for 60.9% of the total number; 4,235,503 and 90,944 SNPs were located in the intron and exon regions, accounting for 36.1% and 0.8% of the total number; and within the exon region, there were 31,112 nonsynonymous mutations and 56,088 synonymous mutations (Tables S1 and S2).

### 2.3. Population Genetic Diversity of Two Breeds

**Figure 2:**
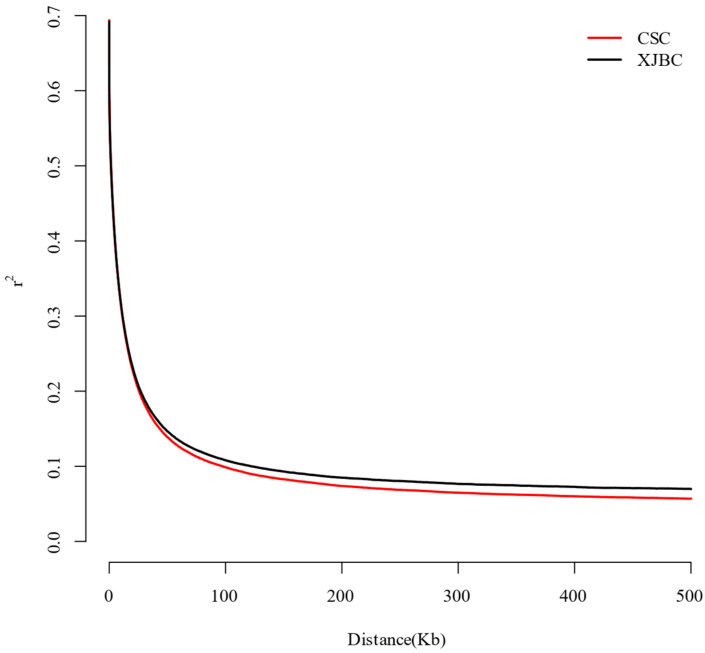
LD attenuation in Xinjiang brown cattle and Chinese Simmental cattle. The horizontal coordinate indicates the distance at which the LD occurred, whereas the vertical coordinate indicates the LD correlation coefficient r^2^.

The population genetic diversity data from Xinjiang brown cattle and Chinese Simmental cattle indicated that the average minimum allele frequencies (MAFs) for the two populations were 0.239 and 0.230, respectively, whereas the average polymorphic information content (PIC) was 0.329 and 0.318, respectively (Table 1). The observed heterozygosity (Ho: 0.335, 0.325) of the two populations was greater than the expected heterozygosity (He: 0.329, 0.318), suggesting that the two populations had high genetic diversity. Figure 2 displays the linkage disequilibrium decay in Xinjiang brown cattle and Chinese Simmental cattle. The average linkage disequilibrium coefficients for the two populations at a 100 kb distance from the genome were 0.1084 and 0.0993, with SNP distances between 0 and 100 kb resulting in faster decay and Chinese Simmental cattle showing faster LD decay than brown cattle. The genetic diversity of Chinese Simmental cattle has declined more rapidly than that of Xinjiang brown cattle, suggesting that the genetic population of Chinese Simmental cattle has a greater level of diversity.

### 2.4. Population Structure Analysis of the Two Breeds

**Figure 3:**
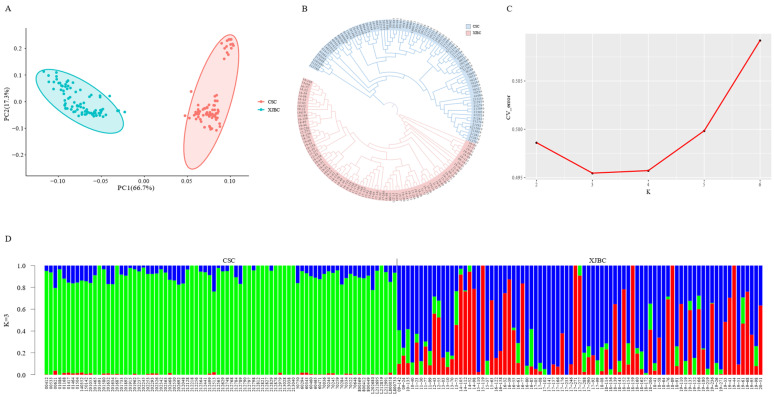
Population structure of Xinjiang brown cattle and Chinese Simmental cattle. (**A**) Principal component analysis of the two breeds. (**B**) Neighbor-joining tree of two breeds. (**C**) Admixture analysis cross-validation error. The abscissa is the K value (2–6), and the ordinate is the cross-validation error. (**D**) Analysis of the population structure of the two populations at K = 3. Every vertical line represents a sample, with the horizontal axis showing the sample number and the vertical axis indicating the percentage of subgroups or ancestors present in each sample. Various colors (blue, green, and red) distinguish between different subgroups or ancestors.

Figure 3A displays the principal component analysis results for the two breeds of cattle, revealing distinct clusters formed by Xinjiang brown cattle and Chinese Simmental cattle, indicating clear population stratification. PC1 and PC2 account for 66.7% and 17.3% of the variance, respectively. To further explore the relationship between the two groups, the neighbor-joining tree constructed by MEGA software is shown in Figure 3B. The results are consistent with the PCA results. The two groups are clustered into groups, and the groups are relatively independent. The genetic structure of the two populations was analyzed using Admixture (Figure 3C). The cross-validation error rate was the lowest when K = 3, making it the optimal K value (Table S3). The hybridization phenomenon of Xinjiang brown cattle was more evident, with a clear separation between the two populations. Some individuals of the two breeds carry genetic components from other ancestral lineages (Figure 3D).

### 2.5. Genetic Distance Matrix and G Matrix Analysis of Two Breeds

**Figure 4:**
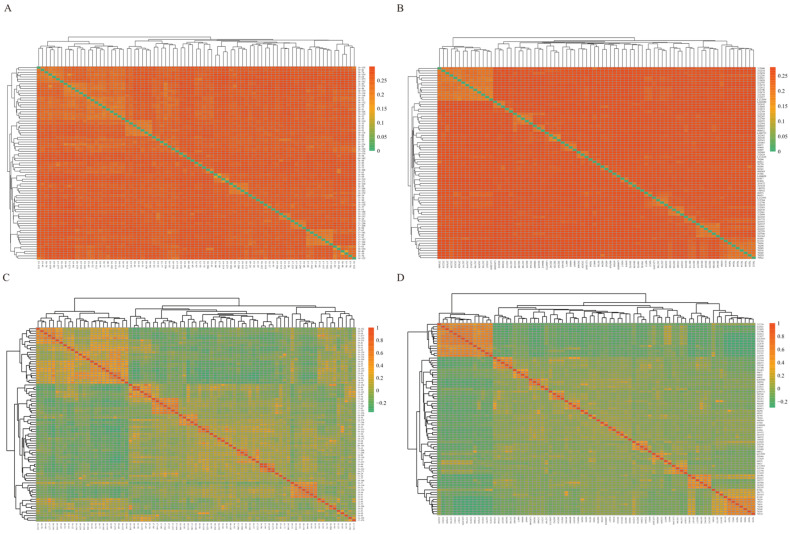
Genetic matrices of Xinjiang brown cattle and Chinese Simmental cattle. (**A**) Genetic distance matrix for Xinjiang brown cattle with IBS. The horizontal and vertical axes represent the individual numbers of the two breeds of cattle, and each small square represents the genetic distance value between two individuals. The closer the color is to orange, the greater the genetic distance; in contrast, the closer the color is to green, the smaller the genetic distance. (**B**) Genetic distance matrix of Chinese Simmental cattle with IBS. (**C**) Kinship G matrix of Xinjiang brown cattle. The horizontal and vertical axes represent the individual numbers of two breeds of cattle, and each small square represents the kinship coefficient between two individuals. The closer the color is to orange, the closer the kinship between individuals is. In contrast, the closer the color is to green, the more distant the kinship is. (**D**) Kinship G matrix of Chinese Simmental cattle.

The IBS distance matrix and the genetic relationship G matrix for Xinjiang brown cattle and Chinese Simmental cattle were constructed using PLINK software. The visualization results of the IBS distance matrix are displayed in Figure 4A,B. The genetic distances based on IBS between most individuals of the two breeds were significant, indicating a moderate genetic relationship, as highlighted by the orange squares in the figure. Additionally, the IBS genetic distances between specific individuals from both breeds were closer, suggesting a closer genetic relationship, as represented by the green squares in the figure. Figure 4C,D illustrate the genetic relationships among individuals. The increasing intensity of color indicates that the genetic relationships are becoming increasingly closer. After conducting a comparative analysis, it was found that the genetic relationship G matrix was consistent with the IBS genetic distance matrix. The genetic relationship was moderate for most individuals from the two breeds, as indicated by the green grid in the figure. However, the genetic relationship was closer for some individuals, as shown by the orange grid in the figure.

### 2.6. Selective Signatures for Milk Production Traits in Two Breeds

#### 2.6.1. Selection Sweep Analysis of Xinjiang Brown Cattle

**Figure 5:**
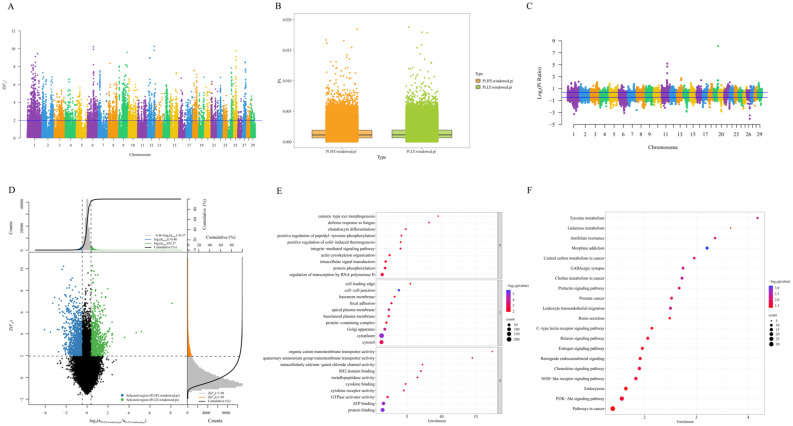
Analysis of selective signatures in Xinjiang brown cattle. (**A**) Manhattan plot of *F*_st_. The blue line represents the threshold value for the top 5% based on the Z(*F*_st_) (1.96). (**B**) PI boxplots of the HY and LY groups. (**C**) Manhattan plot of the θπ ratio. The blue lines represent the threshold value for the top 5% and the below 5% based on the Log_2_ (θπ ratio) (0.37 and −0.46). (**D**) Selective sweep analysis of *F*_st_ and θπ. The Log_2_Pi ratio value is shown on the horizontal axis, whereas the Z(*F*_st_) value is indicated on the vertical axis. The frequency distribution plot is displayed at the top and right, whereas the dot plots in the middle depict the Z(*F*_st_) and Log_2_Pi ratios for various windows. The uppermost blue and green areas represent the top 5% and bottom 5% of the regions selected based on the Log_2_Pi ratio, whereas the orange area on the right indicates the top 5% of the region selected by Z(*F*_st_). The central blue and green areas denote the intersection of Z(*F*_st_) and the Log_2_Pi ratio, which identifies the candidate loci for the HY group and the LY group, respectively. (**E**) GO enrichment analysis of candidate genes identified through the intersection of the two methods. (**F**) KEGG enrichment analysis of candidate genes identified through the intersection of the two methods.

For the analysis of selection signal detection in Xinjiang brown cattle, the whole-genome sliding window method was employed with a window size of 50 kb and a step size of 20 kb. We calculated the fixation index *F*_st_ of the Xinjiang brown cattle population and extracted the top 5% of the Z(*F*_st_) values (Z(*F*_st_) ≥ 1.96) to identify regions under selection (Figure 5A). We calculated the nucleotide diversity values for the HY group and LY group of Xinjiang brown cattle, as illustrated in Figure 5B, which presents box plots of nucleotide diversity for both groups. The nucleotide diversity values for the two groups are clearly similar. We identified selective signals across the entire genome using the θπ ratio in the top and bottom 5% of the regions (Log_2_ (θπ ratio) ≥ 0.37, Log_2_ (θπ ratio) ≤ −0.46) (Figure 5C). Based on the results of the analysis of selection signals using the *F*_st_ and θπ ratios, a selection sweep analysis was employed to detect the selected regions in the HY and LY groups, thereby filtering out candidate genes. The candidate window was identified as the window that appeared in the top 5% region of *F*_st_ and the 5% region before and after the θπ ratio. There were 1617 windows in the selected region of the HY group, including 812 candidate genes. There were 1085 windows in the selected region of the LY group, including 596 candidate genes. Two sets of selection signal detection annotated a total of 1393 candidate genes (Figure 5D).

GO and KEGG enrichment analyses were performed by coannotating the selected regions of the HY and LY groups of Xinjiang brown cattle, resulting in 1393 significant annotations. The results are shown in Figure 5E. The GO enrichment analyses revealed that out of the 1393 candidate genes, 114 GO terms were significantly enriched. Specifically, 59 entries were enriched in BP, 21 in CC, and 34 in MF. The top 10 entries from each category are chosen for presentation. Enrichment analysis revealed that candidate genes in the BP category were enriched in pathways such as the integrin-mediated signaling pathway (GO:0007229, *p* = 3.40 × 10^−4^), protein phosphorylation (GO:0006468, *p* = 0.002522113), and defense response to fungi (GO:0050832, *p* = 0.002625501). Additionally, genes in the CC category were enriched in intercellular junctions (GO:0005911, *p* = 2.30 × 10^−6^), cytoplasm (GO:0005737, *p* = 1.09 × 10^−5^), and Golgi (GO:0005794, *p* = 1.98 × 10^−4^). The MF category was enriched in protein binding (GO:0005515, *p* = 9.12 × 10^−6^), ATP binding (GO:0005524, *p* = 1.64 × 10^−5^), quaternary transmembrane transporter protein activity (GO:0015651, *p* = 2.30 × 10^−4^), and other entries (Table S4). A total of 1393 candidate genes selected from both the HY and LY groups were enriched in 35 KEGG signaling pathways (Figure 5F, Table S6), including the prolactin signaling pathway, the PI3K-Akt signaling pathway, the estrogen signaling pathway, and galactose metabolism, which may be related to milk production performance.

#### 2.6.2. Selection Sweep Analysis of Chinese Simmental Cattle

**Figure 6:**
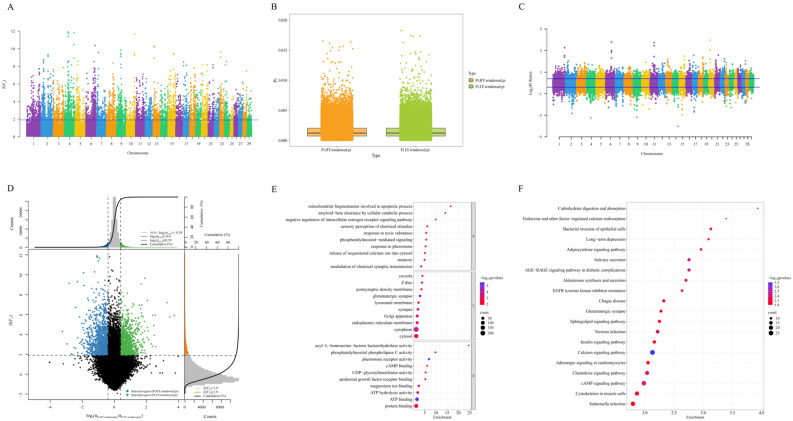
Analysis of selective signatures in Chinese Simmental cattle. (**A**) Manhattan plot of *F*_st_. The blue line represents the threshold value for the top 5% based on the Z(*F*_st_) (1.90). (**B**) PI boxplots of the HY and LY groups. (**C**) Manhattan plot of the θπ ratio. The blue lines represent the threshold value for the top 5% and the below 5% based on the Log_2_ (θπ ratio) (0.39 and −0.40). (**D**) Selective sweep analysis of *F*_st_ and θπ. (**E**) GO enrichment analysis of candidate genes identified through the intersection of the two methods. (**F**) KEGG enrichment analysis of candidate genes identified through the intersection of the two methods.

The whole-genome sliding window method with a window size of 50 kb and a step size of 20 kb was used to detect the selection signal of Chinese Simmental cattle. We calculated the fixation index *F*_st_ of Chinese Simmental cattle and extracted the top 5% Z(*F*_st_) values (Z(*F*_st_) ≥ 1.90) to identify the selected area (Figure 6A). We calculated the nucleotide diversity values of the Chinese Simmental cattle HY group and the LY group separately. Figure 6B shows the box plot of the nucleotide diversity of the two groups. The nucleotide diversity values of the two groups exhibit a degree of similarity. We identified selective signals across the entire genome using the θπ ratio in the top and bottom 5% of the regions (Log_2_ (θπ ratio) ≥ 0.39, Log_2_ (θπ ratio) ≤ −0.40) (Figure 6C). Based on the results of the analysis of selection signals using the *F*_st_ and θπ ratios, a selection sweep analysis was employed to detect the selected regions in the HY and LY groups, thereby filtering out candidate genes. Candidate windows were identified as those appearing in the top 5% region of *F*_st_, as well as the regions 5% before and after the θπ ratio. There were 1502 windows in the selected region of the HY group, including 804 candidate genes. There were 1240 windows in the selected region of the LY group, including 704 candidate genes. Two sets of selection signal detection annotated a total of 1484 candidate genes (Figure 6D).

Enrichment analysis was conducted on 1484 candidate genes from the overlap of the HY and LY groups of Chinese Simmental cattle via the GO and KEGG methods. The GO enrichment analyses revealed that the candidate genes were significantly enriched in 107 GO categories, with 46 entries in BP, 35 entries in CC, and 26 entries in MF. The top 10 entries from each category are chosen for presentation (Figure 6E, Table S5). At the BP level, we enriched memory (GO:0007613, *p* = 0.000376606), sensory perception of chemical stimuli (GO:0007606, *p* = 0.000726934), mitochondrial fragmentation involved in apoptosis (GO:0043653, *p* = 0.001178385), and other terms; at the CC level, we enriched glutamatergic synapses (GO:0098978, *p* = 4.64 × 10^−6^), Z-disk (GO:0030018, *p* = 6.37163 × 10^−5^), and cytoplasm (GO:0005737, *p* = 7.27405 × 10^−5^); and at the MF level, we enriched pheromone receptor activity (GO:0016503, *p* = 2.54925 × 10^−6^), ATP binding (GO:0005524, *p* = 3.79491 × 10^−6^), and phosphatidylinositol phospholipase C activity (GO:0004435, *p* = 0.000234876). The KEGG enrichment analysis shown in Figure 6F revealed that out of the 1484 selected genes, the two groups were enriched in 66 signaling pathways when they overlapped and were significantly enriched in 44 pathways. Among these pathways are the cAMP signaling pathway, insulin signaling pathway, insulin secretion, and ErbB signaling pathway, which may be associated with milk production performance (Table S7).

#### 2.6.3. Distribution of Candidate Genes

**Figure 7:**
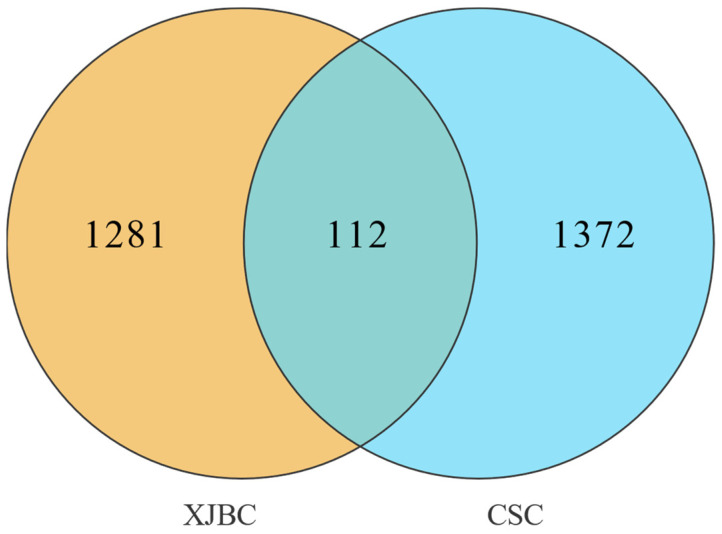
Venn diagram of candidate genes for Xinjiang brown cattle and Chinese Simmental cattle.

By examining the commonality of selection signals and taking the intersection of candidate regions of Xinjiang brown cattle and Chinese Simmental cattle, 112 candidate genes were identified simultaneously (Table S8), as shown in Figure 7. These genes are the focus of attention in the selection process for milk production traits in parturient cattle. Among the genes related to milk yield that have been reported in the literature are *POLB*, *CSGALNACT1*, *DACH1*, *ZFHX3*, *CTNNA3*, and *LAMTOR1*. The genes related to milk protein include *BCAS3*, *NWD2*, *GALNT14*, *TRNAG-UCC*, *TRNAC-ACA*, and *TRNAY-GUA*. The genes associated with milk fat are *METTL15*, *TRNAC-GCA*, *TRNAS-GGA*, *NTRK2*, and *SHC3*, and the genes linked to somatic cell scoring are *TRNAI-AAU* and *TRNAG-CCC*. These genes serve as common key genes in Xinjiang brown cattle and Chinese Simmental cattle.

## 3. Discussion

The main purpose of population genetic diversity analysis is to study the genetic variation within a population and reveal the evolutionary history of species, as well as their environmental adaptability and other key issues. In general, greater genetic diversity within a population leads to increased adaptability to environmental changes and survival capabilities. This not only helps in better understanding species’ evolutionary mechanisms but also serves as a crucial foundation for the sustainable development of genetic resources within populations [18,19]. In this study, we used whole-genome resequencing technology to comprehensively evaluate 80 Chinese Simmental cattle and 83 Xinjiang brown cattle from the perspectives of genetic structure and genetic diversity. We also screened candidate genes related to milk production traits through selection signal analysis, laying the foundation for conservation, utilization, and selective breeding to improve the milk production performance of Xinjiang brown cattle and Chinese Simmental cattle.

The minimum allele frequency is an estimate of alleles that occur less frequently at a population-specific locus [20]. The average minimum allele frequencies in the Xinjiang brown and Chinese Simmental cattle populations of this study were lower than the average minimum allele frequency of 0.28 found in a Chinese population of meat Simmental cattle by Zhu et al. [21]. The proportion of polymorphic information content in the range of 0.25–0.5 was greater in both Xinjiang brown cattle and Chinese Simmental cattle, indicating that most of the SNP loci in the two populations were highly polymorphic. These findings suggest that both populations possess a significant amount of genetic diversity [22]. Heterozygosity is an important indicator of the degree of genetic variation in a population, and its level directly reflects the genetic diversity and adaptive capacity of the population. If the observed heterozygosity exceeds the expected heterozygosity, the population may have historically diverged or imported foreign pedigrees, resulting in greater genetic diversity. Conversely, if the observed heterozygosity falls below the expected heterozygosity, it suggests potential inbreeding within the population [23,24,25]. In this study, we discovered that the observed heterozygosity exceeded the expected heterozygosity in Xinjiang brown cattle and Chinese Simmental cattle. These findings suggest that both breeds have undergone artificial selection, leading to genetic differentiation and high genetic diversity. Linkage disequilibrium analysis can be used to determine diversity differences in a population. A faster LD decay rate indicates that the population is subject to a lower degree of selection and that the genomic genetic diversity is greater [26]. Compared with Xinjiang brown cattle, the Chinese Simmental cattle in this study presented a faster decay rate, suggesting that Chinese Simmental cattle populations presented greater genetic diversity and experienced less genetic selection pressure on their genomes. The slower LD decay observed in Xinjiang brown cattle may be attributed to prolonged intensive selection within the population, which has led to a reduction in adequate population size. The results of the above studies further indicate that Xinjiang brown cattle and Chinese Simmental cattle populations in Xinjiang have high genetic diversity and contain relatively rich genetic resources. These resources are potentially valuable for development and utilization, providing a theoretical basis for the conservation, development, and utilization of the genetic diversity within these two populations.

Evaluating population genetic structure through analyzing SNP data from whole-genome resequencing is crucial for assessing population status [27]. With the continuous expansion of population size, clarifying the kinship relationships between individuals and the population’s genetic structure not only helps to avoid inbreeding but also effectively maintains the genetic diversity of the population, thus promoting the sustainable development of the population. The genetic distance between Xinjiang brown cattle and Chinese Simmental cattle in this study was considerable, indicating a distant kinship relationship and low genetic similarity, reflecting high genetic diversity between the two breeds. The G matrix results corresponded with the IBS genetic matrix results, further verifying the genetic characteristics of the two breeds. The results of PCA and the neighbor-joining tree in this study indicated that the Xinjiang brown cattle and Chinese Simmental cattle populations were independent of each other and had different genetic backgrounds. Admixture analysis of the ancestry components of the populations revealed that the optimal number of ancestors was when K = 3. The genetic structure of the two breeds of cattle was clearly different, with the hybridization phenomenon of Xinjiang brown cattle being more obvious. Most of the individuals had two or three ancestors, possibly because of the infiltration of hybrid genes, leading to the presentation of genetic diversity in the populations.

Mining candidate genes associated with economic traits at the genome-wide level has become an important research tool in the field of animal breeding. *F*_st_ and θπ ratios are effective methods for mining selective signals, revealing differences in allele frequencies within a population, and identifying key molecular markers associated with desirable traits. Combining the two can rapidly screen out gene regions affected by selective pressures and, to a large extent, can avoid false-positive occurrences, pinpointing the functional gene variants that may be involved [28,29]. This study analyzed selective signatures of two breeds to identify candidate genes associated with milk production traits. These genes could serve as important molecular markers for breeding within each breed. Furthermore, by analyzing shared selection signals between the two breeds, the study aims to identify key candidate genes that influence milk production performance in dual-purpose cattle in the Xinjiang region, providing a theoretical basis for the genetic improvement and molecular breeding of local dual-purpose cattle. An investigation of the commonality of selection signals in Xinjiang brown cattle and Chinese Simmental cattle revealed that 112 genes overlapped among the candidate genes in the two breeds of cattle. We conducted a thorough review of the NCBI, GeneCards [30], and the relevant literature to analyze the candidate genes and their biological functions. We found that some genes have been reported to be associated with milk production traits, mainly involved in mammary gland development, milk composition metabolism, and lactation regulation. Firstly, *ZFHX3* interacts with estrogen receptor α and plays a crucial role in mammary gland development, affecting milk yield in dairy cows [31]. Secondly, *CTNNA3* [32], *METTL15* [33], and *NTRK2* [34] are candidate genes affecting milk fat metabolism. *NWD2* [35] and *GALNT14* [36] influence milk protein. *DACH1* [37] may contribute to the regulation of milk cholesterol metabolism. *SHC3* is linked to milk and fat content production in Thai cows of various breeds [38]. *LAMTOR1* [39] converts nonglycans to glucose in the insulin pathway, which regulates glucose metabolism. Furthermore, multiple tRNA clusters, including *TRNAG-CCC*, *TRNAC-GCA*, *TRNAG-UCC*, *TRNAC-ACA*, *TRNAY-GUA*, *TRNAI-AAU*, and *TRNAS-GGA*, are linked to milk production [40]. *POLB* and *CSGALNACT1* influence milk production in Egyptian buffaloes [41]. *BCAS3* impacts milk production in Turkish Holstein cows [42].

Among the candidate genes selected in Xinjiang brown cows and Chinese Simmental cows, the results of the GO and KEGG enrichment analyses of the selected genes revealed significant enrichment in pathways related to milk production. These pathways include the CAMP signaling pathway, glutamatergic synapse pathway, ErbB signaling pathway, insulin signaling pathway, prolactin signaling pathway, PI3K‒Akt signaling pathway, estrogen signaling pathway, endocytosis pathway, and galactose metabolism pathway.

The PI3K/Akt pathway plays a role in the metabolic process of mammary epithelial cell differentiation and milk production in dairy cows through the regulation of glucose uptake and lipid synthesis [43,44]. Candidate genes associated with milk production traits in Thai multibreed dairy cows are enriched in the glutamatergic synaptic, sphingolipid signaling, endocytosis, and ErbB signaling pathways [38]. *GRM8* is associated with 305-day milk yield in Holstein cows and is enriched in the glutamatergic synaptic pathway [45]. The insulin pathway is a metabolic pathway that regulates the metabolism of proteins and lipids, playing a role in controlling glucose‒lipid metabolism [46]. Insulin regulates blood glucose concentration and is essential for milk fat and protein synthesis in dairy cows [47]. The cAMP signaling pathway is linked to milk fat [48]. Jin et al. [49] examined the genetic diversity and selection signals in Dengchuan cattle and discovered a relatively high concentration of genes associated with milk production in the estrogen signaling pathway. The activation of the estrogen signaling pathway leads to extended lactation and increased milk production [50]. Galactose can be found in dairy products as part of the lactose molecule, as well as in glycoproteins, glycolipids, and sugar-containing metabolites, and plays a role in cellular energy production and storage [51,52]. The main hormones that affect the synthesis and secretion of bovine milk proteins are growth hormone, insulin, prolactin, and estrogen [53]. Prolactin (PRL) is a polypeptide hormone produced by various tissues, including the pituitary gland, mammary gland, liver, and gonads. It plays a role in glucose and lipid metabolism, lactation, gonadal functions, and immune system regulation [54,55]. Prolactin was shown by Lacasse et al. [56] to increase the milk production performance of dairy cows. Estrogen is a crucial sex hormone in female animals that controls steroid metabolism, mammary gland growth, and lactation effectiveness [57]. Delbecchui et al. [58] demonstrated that estrogen can control milk production, decrease lactose and milk fat secretion, and increase milk protein content.

## 4. Materials and Methods

### 4.1. Experimental Materials

This study included 83 Xinjiang brown cattle from Yili New Brown Cattle Farm (Yili, China) and 80 Chinese Simmental cattle from Yili Chuangjin Benniu Cattle Animal Husbandry Co., Ltd. (Yili, China). All the research subjects were healthy lactating cattle, and milk production records were gathered for further study. Each individual had 10 mL of blood collected from their tail vein, which was then placed in EDTA anticoagulant tubes and transferred to a sterilized 1.5 mL centrifuge tube. The dry ice was stored and transported to the laboratory to extract DNA.

### 4.2. DNA Extraction and Sequencing

The phenol–chloroform method was used to extract DNA, and 150 bp double-end sequencing was carried out on the DNBSEQ-T7 platform from BGI (MGI Tech, Shenzhen, China).

### 4.3. Alignment and SNP Detection of Sequencing Data

The BWA-MEM algorithm, which is based on BWA v0.7.17 software [59], was used to compare the high-quality clean reads obtained after quality control to the bovine reference genome (ARS-UCD 1.2). The SortSam and MarkDuplicates modules of Picard software v2.25.5 were used for sequencing, and duplicate sequences were removed to minimize repetitive PCR processing. The high-quality comparison results were then analyzed. The HaplotypeCaller, CombineGVCFs, GenotypeGVCFs, SelectVariants, and VariantFiltration modules of GATK v4.4.0.0 software [60] were sequentially used for the detection and screening of raw SNPs. The quality control criteria included QD < 2.0, FS > 60.0, SOR > 3.0, MQ < 40.0, MQRankSum < −12.5, QUAL < 30.0, and ReadPosRankSum < −8.0. The MergeVcfs program of GTAK was used to combine all chromosome VCF files into a genome-wide VCF file. SNP loci were screened with the following retention criteria: (1) minimum allele frequency (MAF) ≥ 0.05; (2) maximum missing rate (MAF) ≤ 0.20; (3) Hardy‒Weinberg equilibrium test *p* value > 1 × 10^−6^; (4) quality score (QUAL) ≥ 30; (5) genotype quality (GQ) ≥ 10; and (6) 2 alleles. Next, VCF files were converted to PLINK format using VCFtools v0.1.17 software [61] to filter out loci with a greater than 5% missing data rate and individuals with more than 10% missing data. The final generated SNPs were compared to the reference genome to annotate the SNP loci using ANNOVAR software v2016-02-01 [62].

### 4.4. Statistical Analysis

#### 4.4.1. Population Genetic Diversity

Genetic diversity analysis was performed on the genomic data of Xinjiang brown cattle and Chinese Simmental cattle after quality control. The expected heterozygosity (He), observed heterozygosity (Ho), minimum allele frequency (MAF), and polymorphic information content (PIC) were calculated by PLINK v1.90 software [63]. PopLDdecay software v3.42 [64] was used to calculate the degree of linkage disequilibrium (LD) decay between SNPs in the populations of Xinjiang brown cattle and Chinese Simmental cattle. The maximum distance was set to 500 base pairs, and a *perl* script was then employed to visualize the LD results and evaluate the genetic diversity of the two populations.

#### 4.4.2. Genetic Distance Matrix and Kinship

To assess the kinship between individuals, we used PLINK software to calculate the genetic distances between individuals of the two populations and analyze populations based on the IBS genetic distances. GCTA v1.94.1 software [65] was used to construct the kinship G-matrices between individuals of the two populations. The IBS genetic distance matrices and kinship G-matrices were then visualized using R v4.4.1 packages (*heatmap*) for both breeds.

#### 4.4.3. Population Genetic Structure

The genetic relationships and genetic distance matrix between the Xinjiang brown cattle and Chinese Simmental cattle populations were analyzed using PLINK software. The R package *ggplot2* was used to visualize the results of the principal component analysis. The *perl* script was used to convert the genetic distance matrix file into a format that MEGA software can use. The neighbor-joining tree was constructed using MEGA v7.0 software [66] using the adjacency method and then visualized using iTOL online software (https://itol.embl.de/, accessed on 21 September 2024). Admixture v1.3.0 software [67] was used to analyze the population genetic structure based on the filtered SNP loci. The population parameter K was established as ranging from 2 to 6, and the cross-validation error rate (CV error) for each K value was calculated individually. By comparing the cross-validation error rates under different K values, the K value with the lowest cross-validation error rate was selected as the best value. The results were visualized using R software.

#### 4.4.4. Population Selection Signatures

The average milk yield between Xinjiang brown cattle and Chinese Simmental cattle was compared under identical milking day conditions. Individuals with phenotypic milk yield records were ranked in descending order based on observed variation, and those lacking phenotypic data were excluded. The individuals were divided into high-yield milk (HY) and low-yield milk (LY) groups via the quartile method. VCFtools software was used to analyze the selection signals in Xinjiang brown cattle and Chinese Simmental cattle populations via the genome-wide sliding window method. The population differentiation index *F*_st_ value and nucleotide polymorphism θπ value were calculated. The window is sorted according to the *F*_st_ value from high to low, and it is corrected to the Z(*F*_st_) value. The ratio of pi.HY/pi.LY was calculated and sorted, and the value of the θπ ratio was normalized using the logarithm to obtain the value of Log_2_ (θπ ratio). The SNP sites in the overlapping windows were annotated using the Bedtools software v2.31.1 [68] based on the reference genome (ARS-UCD 1.2) and annotation files. The formula for calculating Z(*F*_st_) is as follows:

$$ZF_{\mathrm{st}}=\frac{F_{\mathrm{st}}\;\mathrm{value}\;\mathrm{for}\;\mathrm{each}\;\mathrm{window}-\mathrm{the}\;\mathrm{average}\;\mathrm{for}\;\mathrm{all}\;\mathrm{windows}}{\mathrm{Standard}\;\mathrm{deviation}\;\mathrm{for}\;\mathrm{all}\;\mathrm{windows}}$$

The region of the genome showing a strong signal of selective scanning was identified based on the elective sweep analysis using *F*_st_ and θπ values, selecting the window with the top 5% Z(*F*_st_) value and the top 5% and last 5% θπ ratio value. The intersection of the two was then selected to determine the strong selection signal, which is the potential selection region.

#### 4.4.5. GO and KEGG Functional Enrichment Analysis

To enhance the understanding of the molecular function of potential genes, we utilized the David online platform (https://davidbioinformatics.nih.gov/) to conduct GO and KEGG enrichment analyses on genes within overlapping windows. Candidate genes were analyzed for enrichment results in terms of biological process (BP), molecular function (MF), and cellular component (CC) terms. Significant enrichment terms (*p* < 0.05) were selected from all enrichment results. The results were visualized via the website https://www.bioinformatics.com.cn/.

## 5. Conclusions

Both Xinjiang brown cattle and Chinese Simmental cattle populations have rich genetic diversity, but the genetic diversity of Chinese Simmental cattle is greater. The two breeds formed independent clusters with distinct genetic structures, with very few individuals having only one ancestor and most individuals having two or three common ancestors. Based on the fixation index *F*_st_ and nucleotide polymorphism θπ, 112 genes related to milk production traits were screened for selection signals in Xinjiang brown cattle and Chinese Simmental cattle. The insulin signaling pathway, prolactin signaling pathway, PI3K-Akt signaling pathway, and estrogen signaling pathway, which are related to milk production, might play important roles in the selection of milk production traits in the two breeds. This study provides a theoretical foundation for enhancing Xinjiang brown cattle and Chinese Simmental cattle breeds in the future, including breed selection, conservation of genetic resources, and validation of functional genes.

## Figures and Tables

**Table 1:** Genetic diversity indices of Xinjiang brown cattle and Chinese Simmental cattle.

Variety	Minimum Allele Frequency	Polymorphic Information Content	Expected Heterozygosity	Observation of Heterozygosity
Xinjiang brown cattle	0.239	0.329	0.329	0.335
Chinese Simmental cattle	0.230	0.318	0.318	0.325

## Data Availability

The data and material used in this research are available from the corresponding author on request.

## Supplementary Materials

The following supporting information can be downloaded at: https://www.mdpi.com/article/10.3390/ijms26052003/s1.

## References

[1] Zhou J., Liu L., Reynolds E., Huang X., Garrick D., Shi Y. (2021). Discovering copy number variation in dual-purpose Xinjiang brown cattle. Front. Genet..

[2] Xu L., Zhang M., Zhang T., Geng J., Fan S., Yang G., Guo Y., Deng Q., Li J., Liu J. (2023). Genetic parameter estimates for body conformation in Xinjiang brown cattle based on principal component analysis and factor analysis. Acta Vet. Zootech. Sin..

[3] Zhang X., Ge J., Wei C., Zhang M., Wang D., You Z., Huang X., Ma G. (2018). Correlation and regression analysis on body weight and body size index of Simmental calf in Xinjiang area. J. Anim. Ecol..

[4] Xu L., Luo H., Zhang X., Lu H., Zhang M., Ge J., Zhang T., Yan M., Tan X., Huang X. (2022). Factor analysis of genetic parameters for body conformation traits in dual-purpose Simmental cattle. Animals.

[5] Brym P., Kamiński S., Wójcik E. (2005). Nucleotide sequence polymorphism within exon 4 of the bovine prolactin gene and its associations with milk performance traits. J. Appl. Genet..

[6] Zhao Q., Wang L., Ma Y. (2010). Research Progress on QTL mapping of main economic traits in dairy cattle. China Dairy Cattle.

[7] Pedrosa V.B., Schenkel F.S., Chen S.Y., Oliveira H.R., Casey T.M., Melka M.G., Brito L.F. (2021). Genomewide association analyses of lactation persistency and milk production traits in Holstein cattle based on imputed whole-genome sequence data. Genes.

[8] Gangwar M., Ahmad S.F., Gaur G.K., Tamilarasan K., Vyas J., Patel D.A. (2024). Pedigree-based analysis of population structure and genetic diversity in high-milch Vrindavani crossbred cattle of India. Trop. Anim. Health Prod..

[9] Shi Y., Li M., He P., Li F. (2014). Single nucleotide polymorphism and its utilization in plant research. Hans. J. Agric. Sci..

[10] Xia X., Zhang S., Zhang H., Zhang Z., Chen N., Li Z., Sun H., Liu X., Lyu S., Wang X. (2021). Assessing genomic diversity and signatures of selection in Jiaxian Red cattle using whole-genome sequencing data. BMC Genom..

[11] Ma J., Fan A.P., Wang W.S., Zhang J.C., Jiang X.J., Ma R.J., Jia S.Q., Liu F., Lei C.C., Huang Y.Z. (2023). Analysis of genetic diversity and genetic structure of Qinchuan cattle conservation population using whole-genome resequencing. Yi Chuan.

[12] Chen J., Zhang L., Gao L., Wei Z., Dang D., Yang L. (2023). Population structure and genetic diversity of Yunling cattle determined by whole-genome resequencing. Genes.

[13] Chen Q., Zhan J., Shen J., Qu K., Hanif Q., Liu J., Zhang J., Chen N., Chen H., Huang B. (2020). Whole-genome resequencing reveals diversity, global and local ancestry proportions in Yunling cattle. J. Anim. Breed. Genet..

[14] Guan X., Zhao S., Xiang W., Jin H., Chen N., Lei C., Jia Y., Xu L. (2022). Genetic diversity and selective signature in Dabieshan cattle revealed by whole-genome resequencing. Biology.

[15] Wang X., Ma Z., Gao L., Yuan L., Ye Z., Cui F., Guo X., Liu W., Yan X. (2024). Corrigendum: Genome-wide survey reveals the genetic background of Xinjiang Brown cattle in China. Front. Genet..

[16] Ma S., Wang D., Zhang M., Xu L., Fu X., Zhang T., Yan M., Huang X. (2024). Transcriptomic and Metabolomics Joint Analyses Reveal the Influence of Gene and Metabolite Expression in Blood on the Lactation Performance of Dual-Purpose Cattle (*Bos taurus*). Int. J. Mol. Sci..

[17] Zhang T., Li J., Xu L., Wang D., Zhang M., Zhang T., Yan M., Wang W., Fan S., Huang X. (2024). Detection and population structure analysis of genomic structural variation in Xinjiang brown cattle based on whole genome resequencing data. Acta Vet. Zootech. Sin..

[18] Markert J.A., Champlin D.M., Gutjahr-Gobell R., Grear J.S., Kuhn A., McGreevy T.J., Roth A., Bagley M.J., Nacci D.E. (2010). Population genetic diversity and fitness in multiple environments. BMC Evol. Biol..

[19] Bian C., Luo Y., Li J., Cheng H., He F., Duan H., Ahmed Z., Lei C., Yi K. (2024). Inference of Genetic Diversity, Population Structure, and Selection Signatures in Xiangxi White Buffalo of China Through Whole-Genome Resequencing. Genes.

[20] Hu X., You W., Jiang F., Cheng H., Sun Z., Song E. (2025). Analysis of Genetic Diversity and Population Structure of Simmental Cattle Based on Whole Genome Resequencing. Acta Vet. Zootech. Sin..

[21] Zhu M., Zhu B., Wang Y.H., Wu Y., Xu L., Guo L.P., Yuan Z.R., Zhang L.P., Gao X., Gao H.J. (2013). Linkage disequilibrium estimation of Chinese beef Simmental cattle using high-density SNP panels. Asian-Australas. J. Anim. Sci..

[22] Wang P., Ou G., Li G., Li H., Zhao T. (2024). Analysis of genetic diversity and structure of endangered Dengchuan cattle population using a single-nucleotide polymorphism chip. Anim. Biotechnol..

[23] Rajawat D., Ghildiyal K., Sonejita Nayak S., Sharma A., Parida S., Kumar S., Ghosh A.K., Singh U., Sivalingam J., Bhushan B. (2024). Genome-wide mining of diversity and evolutionary signatures revealed selective hotspots in Indian Sahiwal cattle. Gene.

[24] Li Y., Liu L., Zunongjiang A., Cao L., Fan Y., Hu B., Zhang S. (2023). Analysis of the relationship between short tandem repeats and lactation performance of Xinjiang Holstein cows. Trop. Anim. Health Prod..

[25] Schmidt T.L., Jasper M.E., Weeks A.R., Hoffmann A.A. (2021). Unbiased population heterozygosity estimates from genome-wide sequence data. Methods Ecol. Evol..

[26] Xu L., Zhou K., Huang X., Chen H., Dong H., Chen Q. (2024). Whole-genome resequencing provides insights into the diversity and adaptation to desert environment in Xinjiang Mongolian cattle. BMC Genom..

[27] Zhang Y., Wei Z., Zhang M., Wang S., Gao T., Huang H., Zhang T., Cai H., Liu X., Fu T. (2024). Population Structure and Selection Signal Analysis of Nanyang Cattle Based on Whole-Genome Sequencing Data. Genes.

[28] Koloi S., Ganguly I., Singh S., Dixit S. (2025). Whole genome re-sequencing reveals high altitude adaptation signatures and admixture in Ladakhi cattle. Gene.

[29] Rong Y., Jia X., Li P., Tian G., Zhu Z. (2024). Analysis of Genetic Structure Characteristics and Selection Signal in Jinnan Cattle. China Anim. Husb. Vet. Med..

[30] Safran M., Dalah I., Alexander J., Rosen N., Iny Stein T., Shmoish M., Nativ N., Bahir I., Doniger T., Krug H. (2010). GeneCards Version 3: The human gene integrator. Database.

[31] Ooi E., Xiang R., Chamberlain A.J., Goddard M.E. (2024). Archetypal clustering reveals physiological mechanisms linking milk yield and fertility in dairy cattle. J. Dairy Sci..

[32] Cole J.B., Wiggans G.R., Ma L., Sonstegard T.S., Lawlor T.J., Crooker B.A., Van Tassell C.P., Yang J., Wang S., Matukumalli L.K. (2011). Genome-wide association analysis of thirty one production, health, reproduction and body conformation traits in contemporary U.S. Holstein cows. BMC Genom..

[33] Bekele R., Taye M., Abebe G., Meseret S. (2023). Genomic regions and candidate genes associated with milk production traits in Holstein and its crossbred cattle: A review. Int. J. Genom..

[34] Ning M., Zhao Y., Dai D., Yao C., Liu H., Fang L., Wang B., Zhang Y., Cao J. (2023). Gene co-expression network and differential expression analyses of subcutaneous white adipose tissue reveal novel insights into the pathological mechanisms underlying ketosis in dairy cows. J. Dairy Sci..

[35] Lázaro S.F., Tonhati H., Oliveira H.R., Silva A.A., Scalez D.C.B., Nascimento A.V., Santos D.J.A., Stefani G., Carvalho I.S., Sandoval A.F. (2024). Genetic parameters and genome-wide association studies for mozzarella and milk production traits, lactation length, and lactation persistency in Murrah buffaloes. J. Dairy Sci..

[36] Zhou C., Li C., Cai W., Liu S., Yin H., Shi S., Zhang Q., Zhang S. (2019). Genome-Wide Association Study for Milk Protein Composition Traits in a Chinese Holstein Population Using a Single-Step Approach. Front. Genet..

[37] Do D.N., Schenkel F.S., Miglior F., Zhao X., Ibeagha-Awemu E.M. (2018). Genome wide association study identifies novel potential candidate genes for bovine milk cholesterol content. Sci. Rep..

[38] Laodim T., Koonawootrittriron S., Elzo M.A., Suwanasopee T., Jattawa D., Sarakul M. (2024). Genetic factors influencing milk and fat yields in tropically adapted dairy cattle: Insights from quantitative trait loci analysis and gene associations. Anim. Biosci..

[39] Zou N., Wang X., Chen M., Zhang L. (2020). Research progress of *LAMTOR1* in lysosomal metabolism. Biotechnology.

[40] Buaban S., Lengnudum K., Boonkum W., Phakdeedindan P. (2022). Genome-wide association study on milk production and somatic cell score for Thai dairy cattle using weighted single-step approach with random regression test-day model. J. Dairy Sci..

[41] Du C., Deng T., Zhou Y., Ye T., Zhou Z., Zhang S., Shao B., Wei P., Sun H., Khan F.A. (2019). Systematic analyses for candidate genes of milk production traits in water buffalo (*Bubalus bubalis*). Anim. Genet..

[42] Erdoğan M., Çinkaya S., Brenig B., Çelikeloğlu K., Demirtaş M., Sarıibrahimoğlu S., Tekerli M. (2024). Genome-wide association studies for milk production traits and persistency of first calving Holstein cattle in Türkiye. Front. Vet. Sci..

[43] Lin Y., Sun X., Hou X., Qu B., Gao X., Li Q. (2016). Effects of glucose on lactose synthesis in mammary epithelial cells from dairy cow. BMC Vet. Res..

[44] Dbouk H.A., Backer J.M. (2010). A beta version of life: p110β takes center stage. Oncotarget.

[45] Zamorano-Algandar R., Medrano J.F., Thomas M.G., Enns R.M., Speidel S.E., Sánchez-Castro M.A., Luna-Nevárez G., Leyva-Corona J.C., Luna-Nevárez P. (2023). Genetic markers associated with milk production and thermotolerance in Holstein dairy cows managed in a heat-stressed environment. Biology.

[46] Saltiel A.R., Kahn C.R. (2001). Insulin signalling and the regulation of glucose and lipid metabolism. Nature.

[47] Bionaz M., Loor J.J. (2008). Gene networks driving bovine milk fat synthesis during the lactation cycle. BMC Genom..

[48] Kim S., Lim B., Cho J., Lee S., Dang C.G., Jeon J.H., Kim J.M., Lee J. (2021). Genome-wide identification of candidate genes for milk production traits in Korean Holstein cattle. Animals.

[49] Jin L., Qu K., Hanif Q., Zhang J., Liu J., Chen N., Suolang Q., Lei C., Huang B. (2022). Whole-Genome Sequencing of Endangered Dengchuan Cattle Reveals Its Genomic Diversity and Selection Signatures. Front. Genet..

[50] Lawrence R.A. (2022). Breastfeeding.

[51] Battisegola C., Billi C., Molaro M.C., Schiano M.E., Nieddu M., Failla M., Marini E., Albrizio S., Sodano F., Rimoli M.G. (2024). Galactose: A versatile vector unveiling the potentials in drug delivery, diagnostics, and theranostics. Pharmaceuticals.

[52] Iskandar C.F., Cailliez-Grimal C., Borges F., Revol-Junelles A.M. (2019). Review of lactose and galactose metabolism in Lactic Acid Bacteria dedicated to expert genomic annotation. Trends Food Sci. Technol..

[53] Akers R.M. (2006). Major advances associated with hormone and growth factor regulation of mammary growth and lactation in dairy cows. J. Dairy Sci..

[54] Kavarthapu R., Dufau M.L. (2022). Prolactin receptor gene transcriptional control, regulatory modalities relevant to breast cancer resistance and invasiveness. Front. Endocrinol..

[55] Kirsch P., Kunadia J., Shah S., Agrawal N. (2022). Metabolic effects of prolactin and the role of dopamine agonists: A review. Front. Endocrinol..

[56] Lacasse P., Ollier S., Lollivier V., Boutinaud M. (2016). New insights into the importance of prolactin in dairy ruminants. J. Dairy Sci..

[57] Liang J., Shang Y. (2013). Estrogen and cancer. Annu. Rev. Physiol..

[58] Delbecchi L., Miller N., Prud’homme C., Petitclerc D., Wagner G.F., Lacasse P. (2005). 17β-estradiol reduces milk synthesis and increases stanniocalcin gene expression in the mammary gland of lactating cows. Livest. Prod. Sci..

[59] Li H., Durbin R. (2009). Fast and accurate short read alignment with Burrows-Wheeler transform. Bioinformatics.

[60] Nekrutenko A., Taylor J. (2012). Next-generation sequencing data interpretation: Enhancing reproducibility and accessibility. Nat. Rev. Genet..

[61] Danecek P., Auton A., Abecasis G., Albers C.A., Banks E., DePristo M.A., Handsaker R.E., Lunter G., Marth G.T., Sherry S.T. (2011). The variant call format and VCFtools. Bioinformatics.

[62] Wang K., Li M., Hakonarson H. (2010). ANNOVAR: Functional annotation of genetic variants from high-throughput sequencing data. Nucleic Acids Res..

[63] Purcell S., Neale B., Todd-Brown K., Thomas L., Ferreira M.A., Bender D., Maller J., Sklar P., de Bakker P.I., Daly M.J. (2007). PLINK: A tool set for whole-genome association and population-based linkage analyses. Am. J. Hum. Genet..

[64] Zhang C., Dong S.S., Xu J.Y., He W.M., Yang T.L. (2019). PopLDdecay: A fast and effective tool for linkage disequilibrium decay analysis based on variant call format files. Bioinformatics.

[65] Yang J., Lee S.H., Goddard M.E., Visscher P.M. (2011). GCTA: A tool for genome-wide complex trait analysis. Am. J. Hum. Genet..

[66] Kumar S., Stecher G., Li M., Knyaz C., Tamura K. (2018). MEGA X: Molecular evolutionary genetics analysis across computing platforms. Mol. Biol. Evol..

[67] Alexander D.H., Lange K. (2011). Enhancements to the ADMIXTURE algorithm for individual ancestry estimation. BMC Bioinform..

[68] Quinlan A.R., Hall I.M. (2010). BEDTools: A flexible suite of utilities for comparing genomic features. Bioinformatics.

