# Supplementary material for: Analysis of Population Structure and Selective Signatures for Milk Production Traits in Xinjiang Brown Cattle and Chinese Simmental Cattle
Source: Int J Mol Sci. 2025 Feb 25;26(5):2003. doi: 10.3390/ijms26052003 (PMC11900343; doi:10.3390/ijms26052003)
Supplement: Supplementary file 1 [file ijms-26-02003-s001.zip › ijms-3438547-supplementary.pdf]

Analysis of population structure and selective signatures for milk production traits in Xinjiang brown cattle and Chinese Simmental cattle

Supplementary Table S1 Functional classification of the detected SNPs.

Functional classification	Number of SNPs	
	XJBC	CSC
intergenic	6488949	7135370
intronic	3870384	4235503
upstream	61614	70487
downstream	69357	76631
UTR3	70468	79017
UTR5	23240	27368
splicing	301	349
ncRNA	1224	1388
exonic	79249	90944
other	4590	5280

Supplementary Table S2 Functional classification of the exonic SNPs.

Functional classification	Number of SNPs	
	XJBC	CSC
nonsynonymous	25797	31112
synonymous	50169	56088
stopgain	339	393
stoploss	45	54
unknown	2931	3332

Supplementary Table S3 Distribution of cross-validation error values with different K values

K value	CV error
K=2	0.49861
K=3	0.49545
K=4	0.49571
K=5	0.49984
K=6	0.50921

Supplementary Table S4 The top ten significant GO terms from the enrichment analysis of each selected candidate genes of Xinjiang brown cattle

Category	Term	Count	*P*Value	Genes	FDR
BP	GO:0007229~integrin-mediated signaling pathway	11	3.40E-04	TEC,FYB1,ITGA4,SYK,ITGB4,NMRK2,TXK,ITGBL1,PTK2B,CDH17,ITFG1	0.811211
BP	GO:0006468~protein phosphorylation	30	0.002522	ALK,BLK,NIM1K,LRRK1,KALRN,RPS6KA2,PTK2B,MAP3K7,MAP3K5,NTRK2,PDGFRA,MAP2K2,SYK,DAPK2,DAPK3,MOK,MAPK12,TBCK,MAPK11,WEE2,STK17A,KIT,AAK1,BMP2K,POMK,CDK10,SGK1,ANKK1,CDK14,FGFR1	1
BP	GO:0050832~defense response to fungus	5	0.002626	LOC100300237,CATHL4,CLEC6A,LOC538679,ARHGEF40	1
BP	GO:0050731~positive regulation of peptidyl-tyrosine phosphorylation	8	0.002620	GHR,SYK,SEMA4D,CLEC6A,LRRK1,PTK2B,CD44,THBS4	1
BP	GO:0002062~chondrocyte differentiation	7	0.002903	EXT2,OSR1,COL11A2,SLC39A14,GLI3,RUNX2,FGFR1	1
BP	GO:0006357~regulation of transcription by RNA polymerase II	71	0.003503	MESP2,MESP1,ZNF131,IKZF2,GLI3,LOC506181,DACH1,CREB3L3,ZMIZ1,ZNF605,ZNF567,VRTN,NKX3-1,ZNF684,PIAS4,NKX2-8,NKX2-6,OSR1,TSC22D2,SAFB,MED26,FOXP2,SUPT3H,RUNX2,MED6,ETV5,FOXP1,RFX8,ZNF639,TBL1XR1,ZSCAN21,ZNF358,ZNF555,ZNF599,HDGFL2,ZNF554,ZNF276,CEBPA,USP16,CBFB,CEBPG,ZNF3,PRDM11,FOXO3,TBXT,ARNTL,SOHLH2,SAFB2,NKX2-1,BRD9,HIVEP2,ZBTB7A,E2F6,ZKSCAN1,BPTF,ZFHX3,ZNF382,LOC101905588,ZNF181,IRX4,POU6F2,ZBTB10,SMAD9,ELP3,SMAD6,VAX2,ESR1,PHOX2A,ZFP69,TCF4,ZNF333	1
BP	GO:0035556~intracellular signal transduction	24	0.004171	DGKG,NIM1K,SHC3,SYK,DAPK2,DAPK3,LRRK1,MOK,DCDC2,MYO9B,CBLB,ASB18,KALRN,PREX2,MAPK11,TEC,STK17A,RPS6KA2,SPATA13,KIT,RASA2,ARHGEF3,MYZAP,PAG1	1
BP	GO:0030036~actin cytoskeleton organization	13	0.006499	SPECC1,STARD13,ARHGEF18,ANTXR1,SSH1,TBCK,PIK3CA,PALLD,DLC1,KIT,PHACTR2,WASF1,PDLIM4	1

BP	GO:0120162~positive regulation of cold-induced thermogenesis	7	0.006844	DYNC1H1,FABP4,ADRB3,SYK,MFN1,MFN2,DBH	1
BP	GO:0048593~camera-type eye morphogenesis	4	0.007202	RING1,MFN1,MFN2,GLI3	1
CC	GO:0005911~cell-cell junction	19	2.30E-06	MAP2K2,LOC531169,LOC523057,LAMA1,HPN,MAGI2,LOC100298937,RPGRIP1L,LOC512617,LOC100298625,IGSF11,EPB41L3,KIT,KRIT1,CLDN18,FAT3,FAT4,LIMS2,ADGRL3	0.001113
CC	GO:0005737~cytoplasm	211	1.09E-05	HDAC10,PITPNB,CCNF,CBLB,LOC515736,STMN4,SMC2,SPZ1,OGFOD1,ANPEP,RPS6KA2,DZIP1L,DIP2B,ARMT1,MBNL2,CCDC78,DAPK2,DAPK3,MYO7B,MTUS1,MTUS2,WDR72,FNDC3A,ATG12,CD2AP,DDX39A,SPATA17,ULK2,ANKK1,LLGL2,HPN,IQGAP2,KALRN,ERI1,DHX36,BBS2,SPHKAP,FAM136A,BTBD10,GADD45G,SPART,HNRNPM,NOX3,CDK10,DZIP1,CDK14,TECPR2,HBS1L,GLI3,IKBKB,AIFM2,RSRC1,NMRK2,KIF13B,TNFAIP8L1,ARIH1,UBXN6,ANKRD6,DUSP5,MME,SYK,ANXA3,ANXA4,LRRD1,DPYD,MTF2,ARHGEF3,SGK1,PLIN5,PRKN,LOC529488,WDR20,HSD17B8,GNAI1,ARNTL,PTPA,RECQL5,APBB2,CTNNA3,EPS15,SRSF12,WWOX,MTX3,GSX2,LRRC28,MOK,ELP3,VAX2,GTF2H5,ADAT2,NUDT21,PDCL2,FABP4,KIF26B,TTC4,DLC1,TACC1,LIMS2,LOC615989,KLHL31,ARHGAP42,ATCAY,FNTA,BANF2,KPNA2,MAP3K7,KPNA1,PLS1,SH3GL1,PPFIA2,SH3GLB1,HGD,ACOT11,MAGI2,RPSA,ALDH3A1,CCNE2,PPIB,UTRN,HDGFL2,KANK4,KANK3,CSNK1G1,FTO,ANKRD17,KHDRBS1,PSMD12,TEDC2,ISYNA1,TNKS,LRRC51,ARHGAP17,NPAT,ODF2L,TPPP,CCDC141,TBC1D15,ZFHX3,SMAD9,HPS3,ARHGAP28,HOOK3,EEF2,SMAD6,ESR1,SSH1,MOB1B,TBCK,DAB2,KIF18A,DAB1,COPS2,GNB4,TUBGCP6,LSM14A,GALK2,GALK1,RTP4,DOCK5,SH2D4A,LRRK1,PIK3CB,GPHN,PITPNC1,POLB,CEP290,BCAS3,HSP90AA1,SGIP1,DESI2,PLA2G4A,DCK,PIK3CA,BMP2K,ATP6V1B1,NIM1K,GALT,PARG,USP16,DCTN3,USP12,ASB18,TBXT,HAUS8,KATNA1,KRIT1,MROH7,FSD1,WASF1,PDLIM4,TTC12,EYA1,ZNF804A,FBXL16,MAPK12,TTC17,SPATA2L,CAPN14,MAPK11,WEE2,TEC,TCIM,GNPDA2,SPATA33,TRIM35,SARDH,EIF3H,ATXN10,ALDH8A1,SMAP2,PFKM	0.002649
CC	GO:0005794~Golgi apparatus	47	1.98E-04	PRKN,COPB2,GALNT14,CHIC2,GALT,SAR1A,TNKS,PITPNB,INPPL1,FAIM2,HS6ST3,NTN3,MYDGF,SNX1,DACH1,DYM,APBB2,SLC39A7,AP3S1,RAB6A,TMED5,PRMT5,WWOX,PDGFRA,MAP2K2,GOLM1,TMEM30C,DAPK2,PAQR3,MTUS1,P3H2,PDE4DIP,PLA2G4A,HOOK3,FNDC3A,SMAD6,ESR1,IGF2R,RAB11B,EXT2,CCDC170,LOC617224,PXYLP1,CDH15,SERINC5,CD44,ITM2C	0.027569
CC	GO:0016324~apical plasma membrane	24	2.28E-04	SLC22A4,CNTFR,SLC22A5,SLC22A2,ANXA4,SLC22A1,SLC5A11,SLC7A1,SLC39A14,PARD3B,LOC784768,SCNN1B,FXYD1,FAT1,SLC29A4,DPEP1,FOLR3,EZR,PFKM,F2RL2,CD44,SLC19A1,ATP6V1B1,CTSB	0.027569
CC	GO:0005925~focal adhesion	16	7.78E-04	MAP2K2,NEBL,ITGB4,SYNPO2,LPP,THSD1,ARHGAP24,SENP1,PALLD,DLC1,ITGBL1,PTK2B,EZR,WASF1,PHLDB2,LIMS2	0.075341
CC	GO:0005829~cytosol	134	0.001250	LOC615989,DGKG,KRTAP24-1,PANK3,HDAC10,LTN1,INPPL1,CBLB,LOC515736,ABCA12,NFU1,DOCK10,RABGEF1,DACH1,ARHGEF40,KPNA2,PDE8B,VPS36,MAP3K7,PLS1,SH3GLB1,AGTPBP1,ACOT11,DAPK3,P3H2,RPGRIP1L,TICAM1,ALDH3A1,TIAM1,RBP2,RBP1,SPATA13,FKBP8,ULK2,PPIB,EZR,PHLDB2,KANK4,COPB2,TNKS,ANKRD11,TXK,ARHGAP17,FOXO3,ADH6,ADH4,SNX1,COMMD8,DHX36,MAP7,CLCA2,TNRC18,RAB6A,TBCA,SMAD6,EEF2,LOC783466,P4HA2,LOC616199,LOC538679,AAK1,CDK14,GALK2,SERINC5,FGFR1,GALK1,DOCK5,NUMA1,SH2D4A,ELL,LRRK1,CHTF18,GPHN,CALB2,ANKRD9,GHR,GLT1D1,ATIC,EXO5,PHACTR1,PGLS,CEP290,UBXN6,NKX3-1,PRMT5,CCDC39,HSP90AA1,VPS9D1,RING1,SYK,ANXA4,PLA2G4A,ARHGEF18,PALLD,DPYD,PLIN3,PMP2,SGK1,CD44,PLIN5,PRKN,GOT1L1,PARG,PPM1L,SYNPO2,USP12,THSD1,ASB18,RXRB,RECQL5,TP53INP1,ASCC3,XDH,EPS15,NTRK2,SEC24B,FEM1A,AMFR,FABP9,MYO9B,EFR3B,TTC17,MAPK11,FABP4,TCIM,TTC4,DLC1,CCDC6,EIF3H,TACC1,PDE7B,ATXN10,LPIN1,CDO1	0.100794

CC	GO:0016323~basolateral plasma membrane	16	0.003455	SLC22A4,SLC22A5,SLC22A2,SLC22A1,SLC7A1,SLC39A14,SLC4A8,CLDN8,SLC29A4,FOLR3,EZR,SLC12A6,CDH17,CD44,SLC19A1,ATP6V1B1	0.238880
CC	GO:0005604~basement membrane	9	0.006833	COL18A1,FRAS1,LAMA2,LAMA1,ITGB4,P3H2,USH2A,THBS4,NTN3	0.413411
CC	GO:0032991~protein-containing complex	18	0.008750	SH3GLB1,KHDRBS1,HSP90AA1,NUMA1,LOC101905588,CBFB,CLEC12B,SLC7A1,RUNX2,POLB,FKBP8,KRIT1,LALBA,EZR,PPIB,PEX11G,F2RL2,UBXN6	0.470528
CC	GO:0031252~cell leading edge	5	0.011890	ITGB4,LMO4,AAK1,PHLDB2,CD2AP	0.575468
MF	GO:0005515~protein binding	128	9.12E-06	PRDM8,PRDM6,IL23R,CCNF,INPPL1,WDR88,SMC2,KLHL31,PREX2,ARHGAP42,KPNA2,KPNA1,PPFIA2,SH3GL1,SH3GLB1,TLE4,SPINK8,IL11RA,ADIPOQ,P3H2,MYO7B,WDR72,FNDC3A,ANK1,CDC40,TLE6,CD2AP,TIAM2,TIAM1,ARMC2,SPATA17,TBL1XR1,SCN8A,SPATA13,FKBP8,UTRN,ANKK1,KCTD15,KANK4,KANK3,ANKRD17,COPB2,L3MBTL3,RWDD2B,ARHGEF26,C19H17ORF58,TNKS,ANKRD11,TXK,EBI3,TMTC3,TMTC2,LRRC51,ARHGAP17,PRDM11,KALRN,NPAT,WDR70,CASKIN2,VPS8,ZBTB7A,BBS2,STARD13,FYB1,ZBTB10,BTBD10,P4HA2,COPS2,GNB4,ATM,INTS8,TECPR2,ALK,WDR49,SPAG16,COL18A1,TTC23,ZNF131,MEGF11,LRRK1,WDR41,SYNE1,ANKRD9,GHR,PCLO,BOC,UBXN6,ANKRD6,BCAS3,TRPC1,LRRD1,PARD3B,WDR33,LATS1,TLR5,WDR27,WDR20,LRP3,ASB18,NTN3,PDE11A,KRIT1,IL21R,FSD1,CORIN,WWOX,TTC12,FEM1A,LGI4,LRRC28,KLHL2,MYO9B,FBXL16,USH2A,TTC39A,TTC17,FRAS1,LRG1,TEC,TTC6,LOC107131228,DLC1,TRIM35,SARDH,EIF3H,SNTB1,SSBP4,LNX2	0.006736
MF	GO:0005524~ATP binding	88	1.64E-05	ALK,EIF4A2,DGKG,PANK3,LRRK1,CHTF18,ABCA12,ABCA13,GPHN,SMC2,IKBKB,RPS6KA2,LONP1,KIF13B,MAP3K7,MAP3K5,PDGFRA,HSP90AA1,RFC4,MAP2K2,SYK,DAPK2,DAPK3,MYO7B,DDX10,ATP11B,LRRD1,DCK,RUNX2,RAD51B,SELENOO,LATS1,DDX39A,NME6,PIK3CA,KIT,BMP2K,ULK2,SUCLG2,SGK1,ANKK1,ATP6V1B1,CSNK1G1,BLK,SLC22A4,NIM1K,ACSS3,SLC22A5,TXK,KALRN,PTPA,GK5,BTAF1,RECQL5,LOC107131218,KATNA1,DHX36,PTK2B,DPH6,ASCC3,CCT8,LOC100337053,DYNC1H1,NTRK2,ATP8B4,LOC534627,MOK,MYO9B,ACSF3,MAPK12,TBCK,MAPK11,GCLC,KIF18A,TEC,WEE2,STK17A,KIF26B,POMK,AAK1,CDK10,ATM,TRIP13,CDK14,PFKM,GALK2,FGFR1,GALK1	0.006736
MF	GO:0015651~quaternary ammonium group transmembrane transporter activity	5	2.30E-04	SLC44A5,SLC22A4,SLC22A5,SLC22A2,SLC22A1	0.062801
MF	GO:0042169~SH2 domain binding	7	3.67E-04	KHDRBS1,SYK,DLC1,KIT,INPPL1,PAG1,FGFR1	0.075323
MF	GO:0008237~metallopeptidase activity	7	5.63E-04	LOC784768,CLCA2,CLCA1,EIF3H,MPND,CLCA4,CLCA3	0.092379
MF	GO:0015101~organic cation transmembrane transporter activity	4	0.001023	SLC47A2,SLC22A2,SLC22A1,SLC29A4	0.139868
MF	GO:0004896~cytokine receptor activity	8	0.001702	GHR,CNTFR,IL11RA,IL23R,EBI3,IL21R,CD44,IL12RB2	0.199382
MF	GO:0019955~cytokine binding	7	0.002984	GHR,CNTFR,IL11RA,IL23R,EBI3,KIT,IL12RB2	0.305841
MF	GO:0005096~GTPase activator activity	18	0.004042	DOCK5,STARD13,ELMOD1,ARHGAP17,MYO9B,ARHGAP28,IQGAP2,ARHGAP24,PREX2,TBCK,TBC1D2,ARHGAP42,DLC1,RASA2,TBC1D24,SMAP2,TBC1D15,LLGL2	0.306090
MF	GO:0005229~intracellularly calcium-gated chloride channel activity	5	0.004238	LOC784768,CLCA2,CLCA1,CLCA4,CLCA3	0.306090

Supplementary Table S5 The top ten significant GO terms from the enrichment analysis of each selected candidate genes of Chinese Simmental cattle

Category	Term	Count	*P* Value	Genes	FDR
BP	GO:0007613~memory	9	0.000377	TUBA1C,LOC100141266,ITPR3,ITGA5,PTN,PLCB1,CIC,SCN2A,SCN3A	0.786626
BP	GO:0007606~sensory perception of chemical stimulus	7	0.000727	LOC112442216,LOC107131465,LOC112442217,BOSTAUV1R419,BOSTAUV1R418,BOSTAUV1R417,BOSTAUV1R416	0.786626
BP	GO:0043653~mitochondrial fragmentation involved in apoptotic process	4	0.001178	FIS1,ATG3,ERBB4,BAX	0.786626
BP	GO:0019236~response to pheromone	7	0.001525	LOC112442216,LOC107131465,LOC112442217,BOSTAUV1R419,BOSTAUV1R418,BOSTAUV1R417,BOSTAUV1R416	0.786626
BP	GO:0150094~amyloid-beta clearance by cellular catabolic process	4	0.002001	LOC518526,MME,CD36,LOC782615	0.786626
BP	GO:0051209~release of sequestered calcium ion into cytosol	7	0.002115	PLCB4,PLCL2,PLCE1,ITPR3,PLCH1,PLCB1,PLCD4	0.786626
BP	GO:0009636~response to toxic substance	6	0.003361	PON3,PON2,PON1,CYP1B1,BAX,GLYAT	0.937791
BP	GO:0048015~phosphatidylinositol-mediated signaling	6	0.003361	PLCB4,PLCL2,PLCE1,PLCH1,PLCB1,PLCD4	0.937791
BP	GO:0050804~modulation of chemical synaptic transmission	9	0.003963	BCR,PTPRS,PLCB4,PRKAR2B,GRIK4,GRIK2,BTBD9,PPP1R9A,GRIA4	0.982776
BP	GO:0033147~negative regulation of intracellular estrogen receptor signaling pathway	4	0.006266	KANK2,CNOT2,CNOT9,TP63	1
CC	GO:0098978~glutamatergic synapse	25	4.64E-06	PTPRS,HIP1,SEMA3A,SLC1A2,GRIK2,DOCK10,PRKAR2B,ERBB4,SCN3A,EIF4E,DLGAP2,DLGAP4,APPL1,DAPK1,CADPS,ATP2B1,BTBD9,GRIN2C,BCR,DLG1,LRFN5,PLCB4,FILIP1,SCN2A,ADGRL3	0.002391
CC	GO:0030018~Z disc	14	6.37163E-05	NEBL,FHL2,FHL3,NEB,FHL5,JPH1,SYNE2,FBXL22,SMN2,XIRP2,LPXN,MYL9,FBP2,PLEC	0.012487
CC	GO:0005737~cytoplasm	213	7.27405E-05	CYFIP2,LRRC14B,CCNI,WIPF1,PITPNB,PARS2,FRMPD2,DMP1,SLA2,UBASH3B,PPP2R1A,AKT2,LOC782101,KIF21A,BTRC,SKP1,ARMT1,ACOT7,PRKCH,WSB1,DAPK1,PRKCB,SHTN1,SPATA17,HECW2,PNLDC1,HMBS,PADI2,MYL9,NAIP,AIRE,ZNHIT3,APOD,SRPK2,CCR1,TMEM86B,DTNA,DMPK,LNPEP,RMDN2,FHDC1,PARP10,SETX,AGO3,AGO4,AGO1,GIPC2,NOX3,PLCH1,MMS19,GRAP,FARSB,ARF4,ARHGAP8,ARPC1A,SIPA1L2,MEOX2,LRRC1,IKBKB,GYS1,TUBA1C,LOC534181,KIF13A,TNFAIP8L1,CD36,MCF2L2,CCR3,APPL1,MME,TCF12,KIF7,WDR35,DYNLL2,RNF144A,SULT1D1,MDFIC,GAN,PITPNM3,DPYD,GLCCI1,PLCB1,PAFAH1B3,KANK2,CDC123,LOC100141266,NKD2,PSMB7,SCEL,RELN,SULT1E1,EMC2,SPOP,ADAD1,APBB2,CTNNA3,CHML,MAP4K5,MAP4K4,EVC2,DMTN,LOC100299783,STAT3,NLRP13,TTC4,CCNG2,ALMS1,UBAP2,LOC782615,LOC615989,WWC1,CTNND2,CTNND1,RTKN2,FAM107A,RGS2,CAPN5,SVEP1,CMTR1,CDKL3,RALGAPA2,ANKRD44,ACOT11,THEMIS,NOSIP,LOC529196,ACAP2,FRMD6,DMWD,DOK6,SPECC1L,ELMO1,CMPK2,UTRN,VCL,FTL,BRSK1,NDRG3,LOC101902760,LRRC51,AK5,SPATA5,STK33,FGGY,EIF4EBP1,SMN2,SRGAP1,PRPH,AKIRIN2,ZFHX3,NEK4,HSPA6,DHCR24,ARHGAP26,BTBD9,HOPX,FGF17,LIN28B,KIF18A,LOC104973258,CAPRIN1,GNAQ,MAP3K10,GALK2,FGF10,GSK3A,PTPN20,CELF5,PPP1R9A,LOC530538,POLB,DNMT3L,SIN3B,CYP2R1,BCAS3,ACTR2,CAMK1D,UTP11,CKAP5,EVC,TERF2IP,NCBP3,DOCK1,FBP1,FBP2,PAOX,PLEC,HDAC2,PLEKHH2,DCTN1,TGFBRAP1,CRMP1,ASB14,DNAH9,LOC101903496,ASB18,GNA14,ZNRF4,MAPK1,MROH7,RGS22,RAB28,EIF4E,KCNIP4,ZNF804A,MERTK,MAPK10,CAPN13,NELL2,LOC518526,PFKL,WEE2,BAX,VIPAS39	0.012487
CC	GO:0005794~Golgi apparatus	47	0.000473	PPP1R15A,RAB1A,ARF4,CNTNAP2,GALNT14,GALNT13,PARM1,HIP1,PITPNB,ATL1,GALNT18,GLT8D1,HEPACAM2,SLA2,CBL,ZDHHC3,AP3M1,DACH1,CUX1,CTSL,SIX5,FUT9,RXYLT1,DY	0.060952

				M,MAPK1,APBB2,CLSPN,CD36,RAB28,RSAD2,CAV2,ATRNL1,CAV1,LMTK2,FHDC1,PARP10,RNF144A,EXT1,LOC518526,DLG1,GNAQ,RAB18,NUCB1,VIPAS39,ITGA5,GRINA,LOC782615	
CC	GO:0005789~endoplasmic reticulum membrane	46	0.000812	PPP1R15A,DPAGT1,RAB1A,PI4K2B,SAR1B,PITPNB,ATL1,MOGAT1,SEL1L,PIGZ,ITPR3,CYP46A1,JPH1,ZMPSTE24,NCLN,TMEM147,DHRS7B,EMC2,BCAP29,XXYLT1,EMC7,RAB5IF,SEC13,TMEM86B,SEC24A,CERS6,PTGIS,STARD3NL,RSAD2,TMEM178A,SSR3,ACSL6,TAP2,SGPP1,RNF144A,CYP39A1,REEP1,EXT1,DLG1,SPCS1,STIM1,TBXAS1,ACER3,RAB18,BAX,MYORG	0.083649
CC	GO:0005765~lysosomal membrane	21	0.001022	PI4K2B,SEC13,STARD3NL,LOC614531,BRI3,TPCN1,BOLA-DYB,BOLA-DYA,PIP4P2,DSB,LAMP3,LOC539009,VPS11,ATP6V1H,ATP13A2,LAMTOR1,HPSE,GPR155,TECPR1,ATP6V1C2,BOLA-DOB	0.087713
CC	GO:0045202~synapse	25	0.001548	CHRNA1,CYFIP2,RAB1A,STXBP4,RPL34,CHRM5,FAM107A,PPP2CA,CAPN5,SLC12A7,GABRA2,PTPRN2,SLC12A5,DTNA,MME,NPY5R,SLC4A10,SLC6A11,CACNB4,GNAQ,EXOC4,ITGA5,CNTN4,UTRN,CNIH3	0.108579
CC	GO:0005901~caveola	9	0.001687	LOC518526,KIF18A,PTGIS,CAV2,CAV1,MAPK1,CD36,BVES,LOC782615	0.108579
CC	GO:0005829~cytosol	137	0.002147	LOC615989,PI4K2B,WWC1,CTNND1,LTN1,NUDT5,ABCA12,DOCK10,DACH1,BAIAP2L1,SNRPD2,PPP2R1A,AKT2,PLCE1,ANKS1A,TNS3,SKP1,ATG3,PRKCH,ACOT7,COG5,RALGAPA2,ACOT11,EML1,TOM1L1,BCR,PRKAR1B,GAPDHS,ERF,NHLRC1,MASP1,STAM2,RABL6,STXBP2,AK5,THADA,LOC781280,SPATA5,INPP5B,FAM83G,PRKAR2B,SMN2,EIF4EBP1,LPXN,ZNF268,SRPK2,DNAH12,NEK4,OSBPL3,NEK6,HSPA6,INO80,ARHGAP26,PARP10,LIN28B,POLA2,AGO3,AGO4,CAPRIN1,CNOT2,TBXAS1,PLCH1,MMS19,GALK2,DOCK6,PTPRR,GSK3A,PTPRS,ADK,STON2,LOC510369,HK2,LRRC1,TUBA1C,PPP6R1,SIX5,LOC538702,RB1CC1,ZNF385A,APPL1,CEP68,ACTR2,PDE4D,AHSA1,DPYD,TFEC,AHRR,NCBP3,PLIN1,PLCB1,DOCK1,FBP1,FBP2,PLEC,RNF32,PLEKHH2,LOC100141266,DNAH7,DCTN1,NXN,CIPC,AKR1D1,CRMP1,ASB14,MLLT1,CBL,ASB18,PPM1F,SELENBP1,PPP2CA,PSMB7,PSMB2,SULT1E1,VPS50,MXI1,BAK1,ASCC3,CHML,MAP4K5,EIF4E,NTRK2,SEC24A,DMTN,RNF25,TRAPPC10,STAT3,TPRKB,TICRR,AGBL1,FABP6,TTC4,PSMC2,ALMS1,SPAG1,BAX,PLCD4,FRK	0.122879
CC	GO:0098839~postsynaptic density membrane	9	0.003550	DLG1,ERBB4,SYNDIG1,GRIK4,ATP2B2,GRIK2,GRIN2C,SHISA6,GRIA4	0.180287
MF	GO:0016503~pheromone receptor activity	11	2.54925E-06	LOC112442216,LOC107131469,LOC107131465,LOC107131477,BOSTAUV1R414,LOC112442217,LOC104974928,BOSTAUV1R419,BOSTAUV1R418,BOSTAUV1R417,BOSTAUV1R416	0.001478
MF	GO:0005524~ATP binding	95	3.79491E-06	ALK,PI4K2B,TOP2B,GSK3A,MAST4,FBH1,PARS2,ADK,NAT10,ITGAE,ABCA12,ABCA13,HK2,LOC112446383,IKBKB,AKT2,LOC538702,KIF13A,PRKG2,KIF21A,STK32B,EPHA5,MAP2K3,CDKL3,ACTR2,PRKCH,CAMK1D,DNAAF3,DAPK1,PRKCB,MCCC1,ACSL6,TAP2,LMTK2,UBE2E2,DDX10,NAV2,KIF7,BCS1L,RUNX2,BBS12,ABCG4,NAIP,BRSK1,IQCA1,CDC123,DNAH7,ATP10D,ATP1A4,ASB14,ATP1A2,DNAH9,AK5,SPATA5,LOC516849,STK33,BTAF1,ERBB4,MAPK1,TNNI3,ASCC3,MAP4K5,MAP4K4,SRPK2,NTRK2,DNAH12,RRM1,DMPK,UBE2B,NEK4,NEK6,HSPA6,ATP2B2,INO80,ATP2B1,LOC112446470,MERTK,SMARCA2,LOC785370,MAPK10,NLRP13,ATP4A,PFKL,GCLC,KIF18A,WEE2,CAPRIN1,PSMC2,RAD17,MAP3K10,ATP13A2,FRK,GALK2,NEK11,FARSB	0.001478
MF	GO:0004435~phosphatidylinositol phospholipase C activity	6	0.000235	PLCB4,PLCL2,PLCE1,PLCH1,PLCB1,PLCD4	0.060990
MF	GO:0000287~magnesium ion binding	19	0.000988	SRPK2,BRSK1,CDC123,MAST4,DNAAF3,NEK6,ATP10D,LOC510369,LOC516849,ATP4A,GCLC,LOC100125266,LOC534181,WEE2,POLR1A,ALPI,TNNI3,XXYLT1,FARSB	0.169792
MF	GO:0016887~ATP hydrolysis activity	30	0.001090	IQCA1,DNAH7,ATP10D,ATP1A4,ASB14,ATP1A2,DNAH9,ABCA12,SPATA5,ABCA13,LOC516849,BTAF1,KIF13A,KIF21A,ASCC3,DNAH12,HSPA6,TAP2,ATP2B2,NAV2,INO80,ATP2B1,BCS1L,KIF7,ATP4A,PSMC2,RAD17,ATP13A2,ABCG4,NAIP	0.169792
MF	GO:0005515~protein binding	119	0.003886	FRMPD2,SLA2,BAIAP2L1,UBASH3B,PPP2R1A,WDR93,LOC782101,SCMH1,SAMD12,ANKS1A,CMTR1,TNS3,TP63,EPHA5,WSB	0.468783

				1,DAPK1,ANKRD44,OMG,THOC3,TANC2,ACAP2,CACNB4,SPATA17,STIM1,DMWD,PARD3,HECW2,SPECC1L,NHLRC1,ERG,UTRN,KCTD15,LOC615768,IQCA1,NLRX1,SEL1L,WDR64,LRRC51,LOC112449355,FBXO46,ZBTB5,ZNHIT3,CPAMD8,VPS11,CAMTA1,SRGAP1,SCN3A,FIS1,ST14,IQSEC1,FBXO36,ARHGAP26,BTBD9,BTBD8,PHC3,LRFN5,AGO3,AGO4,AGO1,GIPC2,MAP3K10,NOL10,HBP1,GRAP,FBXL5,SCN2A,EIF4G3,ALK,SPAG16,PTPRS,SOWAHB,MAST4,FBH1,TTC22,ASH2L,PTPRK,SIPA1L2,PTPRH,SYNE2,SYNE1,LRRC1,PSD3,BCAS3,SEC13,TTC34,TTC32,PRPF40A,NAV2,WDR35,TMEFF2,GAN,CFAP57,KANK2,RNF32,ASB14,LRP3,ZBTB44,LOC101903496,ASB18,FBXL22,EMC2,UBTD1,PAIP1,PTCD3,ATRNL1,RNF25,LRRC27,TTC9B,MERTK,USH2A,NELL2,NLRP13,FRAS1,PDE10A,KLHL5,KLHL6,ASB4,UBAP2,LGR5	
MF	GO:0008194~UDP-glycosyltransferase activity	6	0.004212	LOC100138004,B3GNT6,LOC540544,GLT8D1,LOC615303,LOC781988	0.468783
MF	GO:0102007~acyl-L-homoserine-lactone lactonohydrolase activity	3	0.004823	PON3,PON2,PON1	0.469674
MF	GO:0030552~cAMP binding	5	0.007820	POPDC3,PDE10A,PRKAR1B,PRKAR2B,BVES	0.676836
MF	GO:0005154~epidermal growth factor receptor binding	5	0.012979	BTC,ERBB4,TGFA,GRAP,SLA2	0.912275

Supplementary Table S6 The top twenty significant KEGG pathways from the enrichment analysis of selected candidate genes of Xinjiang brown cattle

Term	Count	*P*Value	Genes	FDR
bta05032:Morphine addiction	13	0.000666	GABBR2,GABRB1,OPRM1,GNG11,GNAI1,GNG13,GNGT1,GABRR2,PDE11A,GABRR1,GNB4,PDE7B,PDE8B	0.215091
bta05231:Choline metabolism in cancer	12	0.004355	SLC44A5,SLC22A4,DGKG,PDGFRA,MAP2K2,SLC22A5,PIK3CA,SLC22A2,SLC22A1,PLA2G4A,PIK3CB,WASF1	0.417874
bta04670:Leukocyte transendothelial migration	13	0.005616	ITGA4,TXK,PIK3CB,MAPK12,GNAI1,MAPK11,PIK3CA,CLDN8,CLDN18,PTK2B,CTNNA3,CLDN17,EZR	0.417874
bta00350:Tyrosine metabolism	7	0.005823	ALDH3A1,ADH4,GOT1L1,HGD,TOMT,DBH,ADH6	0.417874
bta04727:GABAergic synapse	11	0.006469	GNGT1,GABRR2,GABBR2,GABRR1,GABRB1,LOC529488,GNB4,GNG11,GPHN,GNAI1,GNG13	0.417874
bta01523:Antifolate resistance	8	0.008973	IKBKB,ATIC,LOC107131218,FOLR3,FOLR2,LOC100337053,FOLR1,SLC19A1	0.433297
bta05230:Central carbon metabolism in cancer	9	0.010434	PDGFRA,MAP2K2,PIK3CA,KIT,SLC2A2,SIRT6,PIK3CB,PFKM,FGFR1	0.433297
bta05215:Prostate cancer	11	0.011509	IKBKB,PDGFRA,HSP90AA1,MAP2K2,CREB3L3,PIK3CA,CCNE2,PIK3CB,ETV5,NKX3-1,FGFR1	0.433297
bta04917:Prolactin signaling pathway	10	0.012073	MAPK11,GALT,MAP2K2,SHC3,PIK3CA,SLC2A2,PIK3CB,FOXO3,ESR1,MAPK12	0.433297
bta04062:Chemokine signaling pathway	16	0.021167	LOC508933,SHC3,CCL21,PIK3CB,FOXO3,GNG11,GNAI1,GNG13,GNGT1,IKBKB,TIAM1,PIK3CA,GNB4,PTK2B,CCL19,CCL27	0.683687
bta04151:PI3K-Akt signaling pathway	27	0.02389	LAMA2,LAMA1,ITGB4,PIK3CB,FOXO3,THBS4,GNGT1,GHR,IKBKB,CREB3L3,NTRK2,PDGFRA,HSP90AA1,MAP2K2,ITGA4,SYK,MAGI2,F2R,PPP2R3A,GNG11,GNG13,PIK3CA,CCNE2,KIT,GNB4,SGK1,FGFR1	0.70150
bta04621:NOD-like receptor signaling pathway	16	0.028133	HSP90AA1,LOC112443481,ANTXR2,TICAM1,ANTXR1,MAPK12,ATG12,IKBKB,MAPK11,CATHL4,MFN1,CATHL1,MFN2,MAP3K7,CAMP,CTSB	0.738970
bta04926:Relaxin signaling pathway	12	0.030730	GNGT1,MAPK11,MAP2K2,SHC3,CREB3L3,PIK3CA,GNB4,PIK3CB,GNG11,MAPK12,GNAI1,GNG13	0.738970
bta04723:Retrograde endocannabinoid signaling	13	0.038721	GABRB1,NDUFB4,LOC101906178,GNG11,MAPK12,GNAI1,GNG13,GNGT1,MAPK11,GABRR2,GABRR1,NDUFS6,GNB4	0.738970
bta04924:Renin secretion	8	0.040780	LOC784768,ADRB3,CLCA2,CLCA1,CLCA4,GNAI1,CTSB,CLCA3	0.738970
bta04915:Estrogen signaling pathway	12	0.042379	POMC,GABBR2,HSP90AA1,MAP2K2,SHC3,CREB3L3,PIK3CA,GREB1,OPRM1,PIK3CB,ESR1,GNAI1	0.738970

bta04625:C-type lectin receptor signaling pathway	10	0.043372	IKBKB,MAPK11,CLEC4D,PIK3CA,SYK,CLEC6A,CBLB,PIK3CB,CLEC4E,MAPK12	0.738970
bta00052:Galactose metabolism	5	0.045382	GALT,LOC616199,LALBA,PFKM,GALK1	0.738970
bta05200:Pathways in cancer	34	0.047769	ALK,CEBPA,LAMA2,LAMA1,IL23R,PIK3CB,KNG1,GLI3,GNAI1,GNGT1,RXRB,IKBKB,CTNNA3,IL12RB2,NKX3-1,PDGFRA,HSP90AA1,MAP2K2,DAPK2,DAPK3,NCOA4,F2R,ESR1,GNG11,GNG13,GADD45G,PIK3CA,CCNE2,IFNG,KIT,GNB4,CCDC6,CKS2,FGFR1	0.738970
bta04144:Endocytosis	18	0.048014	SH3GLB1,PDGFRA,CBLB,IGF2R,RAB11B,SPART,SNX4,DAB2,SNX1,CHMP1A,FOLR3,SMAP2,RAB11FIP2,FOLR2,FOLR1,VPS36,EPS15,SH3GL1	0.738970

Supplementary Table S7 The top twenty significant KEGG pathways from the enrichment analysis of selected candidate genes of Chinese Simmental cattle

Term	Count	*P* Value	Genes	FDR
bta04020:Calcium signaling pathway	26	4.984376334179903E-4	CHRM5,TGFA,PDGFA,ITPR3,CACNA1I,GNA14,EDNRB,SLN,ERBB4,PLCE1,NTRK2,CAMK1D,PRKCB,ATP2B2,ATP2B1,TACR1,GRIN2C,TPCN1,FGF17,PLCB4,GDNF,STIM1,GNAQ,PLCB1,PLCD4,FGF10	0.148534
bta05100:Bacterial invasion of epithelial cells	11	0.002447	ACTR2,SHC3,CAV2,CAV1,ARPC1A,ELMO1,CTNNA3,ITGA5,CBL,DOCK1,VCL	0.170166
bta04933:AGE-RAGE signaling pathway in diabetic complications	13	0.002488	EDN1,CXCL8,PRKCB,STAT3,MAPK10,PLCB4,AKT2,NOX4,PLCE1,MAPK1,BAX,PLCB1,PLCD4	0.170166
bta04970:Salivary secretion	13	0.002488	LOC787241,LYZ3,PRKCB,ATP1A4,ATP2B2,ITPR3,ATP1A2,ATP2B1,PLCB4,GNAQ,PRKG2,PLCB1,LOC617219	0.170166
bta04024:cAMP signaling pathway	23	0.002855	POPDC3,EDN1,GIPR,PDE4D,ATP1A4,NPY1R,ATP2B2,GCG,ATP1A2,ATP2B1,GRIN2C,MAPK10,PDE10A,HCAR1,AKT2,NPY,PLCE1,MAPK1,GHRL,TNNI3,MYL9,BVES,GRIA4	0.170166
bta04973:Carbohydrate digestion and absorption	8	0.003689	PLCB4,PRKCB,AKT2,SI,ATP1A4,ATP1A2,PLCB1,HK2	0.183220
bta04925:Aldosterone synthesis and secretion	12	0.004554	CACNA1I,CAMK1D,PLCB4,STAR,PRKCB,GNAQ,ATP1A4,ATP2B2,ITPR3,ATP1A2,ATP2B1,PLCB1	0.193853
bta05135:Yersinia infection	16	0.005547	MAP2K3,ACTR2,CXCL8,WIPF1,ARPC1A,IL2,MAPK10,IKBKB,FCGR2A,CD8B,AKT2,GNAQ,ELMO1,MAPK1,ITGA5,DOCK1	0.203428
bta04920:Adipocytokine signaling pathway	10	0.006144	MAPK10,IKBKB,LOC518526,AKT2,NPY,STAT3,SLC2A1,ACSL6,CD36,APPL1	0.203428
bta04062:Chemokine signaling pathway	18	0.007088	CCR1,GSK3A,CXCL8,SHC3,PRKCB,STAT3,LOC529196,CXCL5,IKBKB,PLCB4,AKT2,GNAQ,PARD3,ELMO1,MAPK1,PLCB1,CCR3,CCL26	0.209982
bta04730:Long-term depression	9	0.008025	PPP2CA,PLCB4,PPP2R1A,PRKCB,GNAQ,PRKG2,MAPK1,ITPR3,PLCB1	0.209982
bta04961:Endocrine and other factor-regulated calcium reabsorption	8	0.008456	PLCB4,PRKCB,GNAQ,ATP1A4,ATP2B2,ATP1A2,ATP2B1,PLCB1	0.209982
bta05142:Chagas disease	13	0.009839	CXCL8,FASLG,IL2,PPP2CA,MAPK10,IKBKB,GNA14,PLCB4,PPP2R1A,AKT2,GNAQ,MAPK1,PLCB1	0.213696
bta04820:Cytoskeleton in muscle cells	20	0.010857	FBN3,DTNA,NEBL,ATP1A4,FHL2,FHL3,NEB,ATP1A2,SYNE2,SYNE1,SGCD,TNNT1,DAAM2,XIRP2,TNNI3,COL9A2,ITGA5,MYL9,VCL,PLEC	0.213696
bta05132:Salmonella infection	22	0.010867	MAP2K3,CYFIP2,ACTR2,LOC100141266,CXCL8,DCTN1,TCF7,ARPC1A,DYNLL2,MAPK10,IKBKB,TUBA1C,AKT2,EXOC4,VPS11,ELMO1,MAPK1,BAX,BAK1,MYL9,NAIP,SKP1	0.213696
bta04910:Insulin signaling pathway	14	0.012494	SHC3,CBL,HK2,MAPK10,IKBKB,GYS1,PRKAR1B,PRKAR2B,AKT2,EIF4EBP1,MAPK1,FBP1,EIF4E,FBP2	0.213696
bta04071:Sphingolipid signaling pathway	13	0.012565	CERS6,PRKCB,SGPP1,PPP2CA,MAPK10,PLCB4,SPTLC2,PPP2R1A,AKT2,GNAQ,MAPK1,BAX,PLCB1	0.213696
bta01521:EGFR tyrosine kinase inhibitor resistance	10	0.012908	SHC3,PRKCB,AKT2,STAT3,EIF4EBP1,PDGFA,TGFA,MAPK1,BAX,EIF4E	0.213696

bta04261:Adrenergic signaling in cardiomyocytes	15	0.014328	POPDC3,ATP1A4,ATP2B2,ATP1A2,ATP2B1,PPP2CA,PLCB4,CACNB4,PPP2R1A,AKT2,GNAQ,MAPK1,TNNI3,PLCB1,BVES	0.224723
bta04724:Glutamatergic synapse	12	0.01591089	PLCB4,PRKCB,GNAQ,GRM8,SLC1A2,GRIK4,MAPK1,ITPR3,GRIK2,PLCB1,GRIN2C,GRIA4	0.237072

Supplementary Table S8 Two breeds share common genes

Gene	Descriptions
LOC112448311	Small Nucleolar RNA SNORA70
MME	Membrane Metalloendopeptidase
LOC104970303	Uncharacterized LOC104970303
LTN1	Listerin E3 Ubiquitin Protein Ligase 1
GALK2	Galactokinase 2
GALNT14	Polypeptide N-Acetylgalactosaminyltransferase 14
ALK	ALK Receptor Tyrosine Kinase
LOC112448818	Uncharacterized LOC112448818
NEBL	Nebulette
TRNAE-UUC	Transfer RNA Glutamic Acid (Anticodon UUC)
LOC107133045	Uncharacterized LOC107133045
JPH1	Junctophilin 1
TRNAG-CCC	Transfer RNA Glycine (Anticodon CCC)
TRNAC-GCA	Transfer RNA Cysteine (Anticodon GCA)
LRRC51	Leucine Rich Repeat Containing 51
LAMTOR1	Late Endosomal/Lysosomal Adaptor, MAPK And MTOR Activator 1
TOMT	Transmembrane O-Methyltransferase
ANAPC15	Anaphase Promoting Complex Subunit 15
TRNAG-UCC	Transfer RNA Glycine (Anticodon UCC)
LOC617507	Olfactory Receptor Family 5 Subfamily B Member 147, Pseudogene
LOC784993	Olfactory Receptor Family 5 Subfamily B Member 136, Pseudogene
LOC506652	Olfactory Receptor Family 5 Subfamily B Member 12C
LOC784897	Olfactory Receptor Family 5 Subfamily B Member 148
USH2A	Usherin
TRNAC-ACA	Transfer RNA Cysteine (Anticodon ACA)
LRP3	LDL Receptor Related Protein 3
SLC7A10	Solute Carrier Family 7 Member 10
CEBPG	CCAAT Enhancer Binding Protein Gamma
PEPD	Peptidase D
KCTD15	Potassium Channel Tetramerization Domain Containing 15
BCAS3	BCAS3 Microtubule Associated Cell Migration Factor
SPAG16	Sperm Associated Antigen 16
ABCA12	ATP Binding Cassette Subfamily A Member 12
ZNF804A	Zinc Finger Protein 804A
TRNAY-GUA	Transfer RNA Tyrosine (Anticodon GUA)
TRNAI-AAU	Transfer RNA Isoleucine (Anticodon AAU)
TRNAT-AGU	Transfer RNA Threonine (Anticodon AGU)
TRNAS-GCU	Transfer RNA Serine (Anticodon GCU)
IKBKB	Inhibitor Of Nuclear Factor Kappa B Kinase Subunit Beta
POLB	DNA Polymerase Beta
CSGALNACT1	Chondroitin Sulfate N-Acetylgalactosaminyltransferase 1
CTNNA3	Catenin Alpha 3
ASB18	Ankyrin Repeat And SOCS Box Containing 18
DPYD	Dihydropyrimidine Dehydrogenase
KIAA1147	DENN Domain Containing 11
WEE2	WEE2 Oocyte Meiosis Inhibiting Kinase
COBL	Cordon-Bleu WH2 Repeat Protein
FOXP2	Forkhead Box P2
TRNAS-GGA	Transfer RNA Serine (Anticodon GGA
LOC101904412	Thymidylate synthase-like
ARHGAP24	Rho GTPase Activating Protein 24
ADGRL3	Adhesion G Protein-Coupled Receptor L3
LOC781649	UDP-glucuronosyltransferase 2B4-like
TRNAA-UGC	Transfer RNA Alanine (Anticodon UGC)
TNFAIP8L1	TNF Alpha Induced Protein 8 Like 1
NTRK2	Neurotrophic Receptor Tyrosine Kinase 2
LOC100335718	SREBP regulating gene protein-like
HS3ST5	Heparan Sulfate-Glucosamine 3-Sulfotransferase 5

UTRN	Utrophin
SYNE1	Spectrin Repeat Containing Nuclear Envelope Protein 1
NOX3	NADPH Oxidase 3
C1H3orf52	Chromosome 3 Open Reading Frame 52
GCSAM	Germinal Center Associated Signaling And Motility
CCDC169	Coiled-Coil Domain Containing 169
LOC784243	Large ribosomal subunit protein eL34-like
SOHLH2	Spermatogenesis And Oogenesis Specific Basic Helix-Loop-Helix 2
DACH1	Dachshund Family Transcription Factor 1
DDX10	DEAD-Box Helicase 10
KIF18A	Kinesin Family Member 18A
METTL15	Methyltransferase 15, Mitochondrial 12S RRNA N4-Cytidine
SPATA17	Spermatogenesis Associated 17
LOC112441820	Uncharacterized LOC112441820
CNIH3	Cornichon Family AMPA Receptor Auxiliary Protein 3
EFCAB2	EF-Hand Calcium Binding Domain 2
PITPNB	Phosphatidylinositol Transfer Protein Beta
LOC101903015	Uncharacterized LOC101903015
ZFHX3	Zinc Finger Homeobox 3
TRNAK-UUU	Transfer RNA Lysine (Anticodon UUU)
SPECC1	Sperm Antigen With Calponin Homology And Coiled-Coil Domains 1
DOCK10	Dedicator Of Cytokinesis 10
LOC112442542	uncharacterized LOC112442542
SEMA5A	Semaphorin 5A
IRX4	Iroquois Homeobox 4
NDUFS6	NADH:Ubiquinone Oxidoreductase Subunit S6
MRPL36	Mitochondrial Ribosomal Protein L36
FAM19A4/TAFA4	TAFA Chemokine Like Family Member 4
RUNX2	RUNX Family Transcription Factor 2
GCLC	Glutamate-Cysteine Ligase Catalytic Subunit
DYM	Dymeclin
FGFBP3	Fibroblast Growth Factor Binding Protein 3
BTAF1	B-TFIID TATA-Box Binding Protein Associated Factor 1
TRNAD-GUC	Transfer RNA Aspartic Acid (Anticodon GUC)
TTC4	Tetratricopeptide Repeat Domain 4
MROH7	Maestro Heat Like Repeat Family Member 7
FAM151A	Family With Sequence Similarity 151 Member A
LOC112446164	U6 Spliceosomal RNA
LOC615989	Protein FAM151A
TRNAP-AGG	Transfer RNA Proline (Anticodon AGG)
ACOT11	Acyl-CoA Thioesterase 11
ABCA13	ATP Binding Cassette Subfamily A Member 13
COA1	Cytochrome C Oxidase Assembly Factor 1
OSTC	Oligosaccharyltransferase Complex Non-Catalytic Subunit
RPL34	Ribosomal Protein L34
NWD2	NACHT And WD Repeat Domain Containing 2
APBB2	Amyloid Beta Precursor Protein Binding Family B Member 2
FRAS1	Fraser Extracellular Matrix Complex Subunit 1
SHC3	SHC Adaptor Protein 3
GRIK2	Glutamate Ionotropic Receptor Kainate Type Subunit 2
ASCC3	Activating Signal Cointegrator 1 Complex Subunit 3
RMND1	Required For Meiotic Nuclear Division 1 Homolog
ARMT1	Acidic Residue Methyltransferase 1
LOC112448082	Uncharacterized LOC112448082
